# Efficacy of systemic oncological treatments in patients with advanced esophageal or gastric cancers at high risk of dying in the middle and short term: an overview of systematic reviews

**DOI:** 10.1186/s12885-021-08330-5

**Published:** 2021-06-16

**Authors:** M. Santero, J. Pérez-Bracchiglione, R. Acosta-Dighero, A. G. Meade, A. Antequera, A. Auladell-Rispau, M. J. Quintana, C. Requeijo, G. Rodríguez-Grijalva, K. Salas-Gama, R. Dorantes-Romandia, J. Salazar, I. Solà, G. Urrútia, X. Bonfill Cosp

**Affiliations:** 1 Iberoamerican Cochrane Centre, Biomedical Research Institute Sant Pau (IIB Sant Pau), C/ Sant Antoni Maria Claret, 167, Pavelló 18, planta 0, 08025 Barcelona, Spain; 2 Interdisciplinary Centre for Health Studies (CIESAL), Universidad de Valparaíso, Viña del Mar, Chile; 3 School of Physiotherapy, Faculty of Health Sciences, Universidad San Sebastian, Santiago, Chile; 4 CIBER Epidemiología y Salud Pública (CIBERESP), Universitat Autònoma Barcelona, Barcelona, Spain

**Keywords:** Esophageal Cancer, Gastric Cancer, Antineoplastic agents, Biological therapy, Molecular targeted therapy, Immunotherapy, Review literature as topic, Systematic reviews

## Abstract

**Background:**

Esophageal and gastric cancers are a significant public health problem worldwide, with most patients presenting with advanced-stage disease and, consequently, poor prognosis. Systemic oncological treatments (SOT) have been widely used over more conservative approaches, such as supportive care. Nevertheless, its effectiveness in this scenario is not sufficiently clear. This paper provides an overview of systematic reviews that assessed the effectiveness of SOT compared with the best supportive care (BSC) or placebo in patients with advanced esophageal or gastric cancers in an end-of-life context.

**Methods:**

We searched MEDLINE, EMBASE, The Cochrane Library, Epistemonikos, and PROSPERO for eligible systematic reviews (SRs) published from 2008 onwards. The primary outcomes were overall survival (OS), progression-free survival (PFS), functional status, and toxicity. Two authors assessed eligibility and extracted data independently. We evaluated the methodological quality of included SRs using the AMSTAR-2 tool and the overlap of primary studies (corrected covered area, CCA). Also, we performed a de novo meta-analysis with data reported for each primary study when it was possible. We assessed the certainty of evidence using the GRADE approach.

**Results:**

We identified 16 SRs (19 included trials) for inclusion within this overview. Most reviews had a critically low methodological quality, and there was a very high overlap of primary studies. It is uncertain whether SOT improves OS and PFS over more conservative approaches due to the very low certainty of evidence.

**Conclusions:**

The evidence is very uncertain about the effectiveness of SOT for advanced esophageal or gastric cancers. High-quality SRs and further randomized clinical trials that include a thorough assessment of patient-centered outcomes are needed.

**Trial registration:**

Open Science Framework, 10.17605/OSF.IO/7CHX6.

**Supplementary Information:**

The online version contains supplementary material available at 10.1186/s12885-021-08330-5.

## Background

Worldwide, esophageal and gastric cancers are a significant public health problem, with approximately 509,000 and 783,000 deaths in 2018, respectively [1]. Their combined mortality for both tumor sites is over 1.2 million, leading to the second most common cancer death cause after lung cancer. While global reports have shown a decrease in gastric cancer mortality rates over the past 20 years, a steady increase in esophageal cancer rates has been observed mainly in the Western Pacific and European regions [2]. Moreover, both cancers are overly aggressive; despite their relatively low incidence, they often have a poor prognosis since the diagnosis is usually late [3, 4]. In a metastatic stage, esophageal and gastric cancers have less than 30% survival at 1 year and less than 5% at 5 years, respectively [5]. Due to the above, many patients are in a terminal care period with progressive disease and months or less of expected survival which has been conceptualized by some authors as “end of life” (EOL) [6, 7].

The use of systemic oncological treatments (SOT) has been widely investigated for esophageal and gastric cancers, and as a consequence, chemotherapy (CT), targeted therapy, and immunotherapy are largely used to try to improve survival and quality of life (QoL) [8, 9]. However, its use in the EOL context is still subject to controversy. Some authors have reported patients experiencing emotional distress, severely reduced QoL, a range of diagnosis-specific and treatment-related problems, and side effects related to these treatments [10, 11]. The overuse of SOT close to death could be an indicator of low-quality medical care, defined as the underuse of known effective practices, or equivocal effectiveness according to the provider rather than patient preferences [12].

More knowledge is needed to improve the ability of the current healthcare system to deliver timely and appropriate EOL care. Among patients with esophageal or gastric cancers with poor prognosis, a palliative care approach is imperative [13]. In this sense, best supportive care (BSC) may include a range of multidisciplinary interventions, such as symptomatic control by radiotherapy, palliative surgery, management of antineoplastic-treatment-related toxicities, analgesia, and psychological or social assistance [13–15].

It would be very useful to know the precise balance of whether these effective treatments compensate for the adverse effects and costs they have for patients and society. Therefore, it is of central importance to evaluate the appropriateness of the SOT compared to the existing alternatives, such as BSC, in terms of effectiveness with special consideration for the patient’s QoL near death and relief of the significant physical and psychological symptomatic burden that these patients present. Thus, this study aims to make a comprehensive synthesis of the available evidence regarding the effectiveness of SOT from systematic reviews (SRs) compared with BSC or placebo in patients with advanced esophageal or gastric cancers in an EOL context.

## Methods

We performed an overview of SRs on patients with advanced esophageal or gastric cancers published from 2008 onwards. The current study is part of a broader evidence syntheses project that aims to assess the effectiveness of SOT versus BSC for patients with advanced non-intestinal digestive cancer (esophageal, gastric, hepatobiliary, and pancreatic cancer). We registered the protocol detailing the methods in the Open Science Framework (see protocol in Additional file 1) [16] and we conducted this overview according to rigorous standards aligned to Cochrane Methodology [17] and reported our results according to PRISMA (Preferred Reporting Items for Systematic Reviews and Meta-Analyses) guidelines [18] (see the completed checklist in Additional file 2).

### Search strategy and selection criteria

**Fig. 1:**
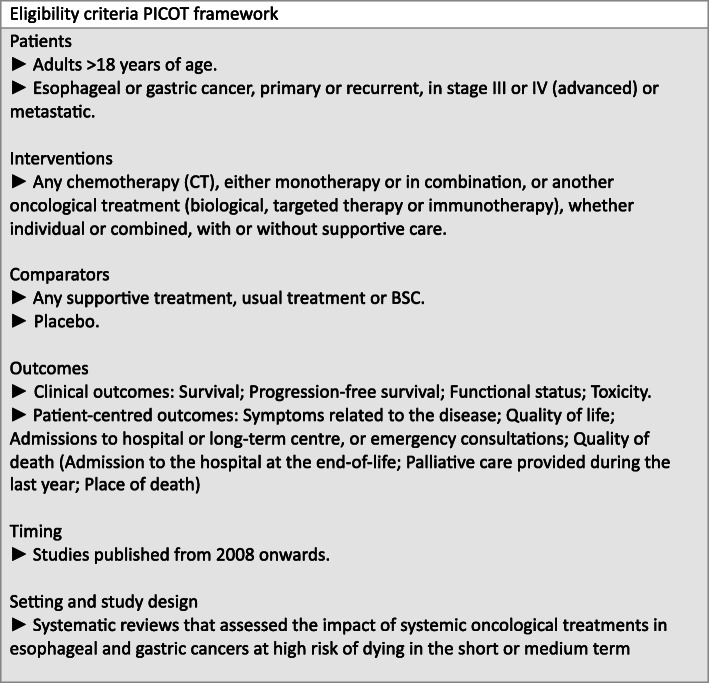
Eligibility criteria

Figure 1 presents our eligibility criteria. We included SRs that assessed SOT’s impact in esophageal or gastric cancer patients at high risk of dying in the short or medium term. We searched for the following outcomes: 1) overall survival (OS); 2) progression-free survival (PFS); 3) functional status (FS); 4) toxicity; 5) symptoms related to the disease; 6) QoL; 7) admissions to hospital or long-term center, or emergency consultations; 8) quality of death (admission to the hospital at the end-of-life; palliative care provided during the last year; place of death). We considered the following as primary outcomes: OS, QoL, FS, and toxicity.

We searched in four bibliographic databases: MEDLINE (access via PubMed), the Cochrane Database of Systematic Reviews, Epistemonikos from inception to September 30th, 2019, and EMBASE (access via Ovid) from inception to October 7th, 2019. We did not restrict our search by language. We provide a detailed search strategy elsewhere [16]. The search strategy for PubMed is described in Additional file 3. Two previously trained reviewers performed an independent title and abstract screening and a full-text screening afterward. A third reviewer solved any disagreements. We used Covidence for all the screening process [19].

### Data extraction and Risk of Bias Assessment

One reviewer extracted data from the included studies using a previously piloted data extraction sheet, and a second author cross-checked this process. We extracted from the included SRs both synthesized findings and disaggregated data on reported outcomes of interest for each primary study. One author assessed the methodological quality for each SR using the A MeaSurement Tool to Assess Systematic Reviews (AMSTAR-2) tool, and a second author cross-checked this assessment [20]. We reported the risk of bias assessment of primary studies undertaken by the authors of each SR. When two or more SRs had a conflicting risk of bias assessments for a primary study, we reported the one assessed by the Cochrane tool. If disagreement persisted, we reported the assessment of the SR with better methodological quality according to AMSTAR-2 (if the reviews had the same quality, we selected the most frequent judgment from the primary study assessment). Lastly, if a discrepancy remained, we reported it as “no agreement”.

### Assessment of overlap of primary studies

We built a matrix of evidence to assess the overlap of primary studies within SRs. We computed the matrix cross-linking the relevant randomized control trials (RCTs) in eligible SRs for this overview and calculated the corrected covered area (CCA). We considered a CCA below 5% as slight overlap, a CCA > 5 and < 10% as moderate overlap, a CCA > 10 and < 15% as high overlap, and a CCA > 15% as a very high overlap [21].

### Data synthesis and analysis

We presented a narrative synthesis of the included reviews and summarised the main results on the effectiveness of SOT regarding relevant outcomes. We performed a de novo meta-analysis based on primary studies data included in eligible SRs when possible for each comparison. We analyzed dichotomous outcomes with an odds ratio (OR), continuous outcomes with the mean difference or standardized mean difference, and time-to-event outcomes with hazard ratios (HR), all of these with a 95% confidence interval (95% CI). We assessed the heterogeneity of the included studies with I^2^ as follows: I^2^ < 50% as low heterogeneity, I^2^ > 50 and < 90% as high, and > 90% as very high. When heterogeneity was below 90%, we performed a meta-analysis in RevMan 5.4 using a random-effects model. We reported all the outcomes according to a type of SOT (chemotherapy, immunotherapy, and targeted/biological therapies). We also conducted a sensitivity analysis, considering only studies in which comparison is described explicitly as BSC.

### Assessment of certainty of the evidence

We assessed the certainty of the evidence for each primary outcome according to the Grading of Recommendations Assessment, Development, and Evaluation (GRADE) guidance and performed a Summary of Findings (SoF) table [22]. We classified the certainty of the evidence for each outcome as high, moderate, low, or very low. We also reported the SoF in plain-language summary.

## Results

**Fig. 2:**
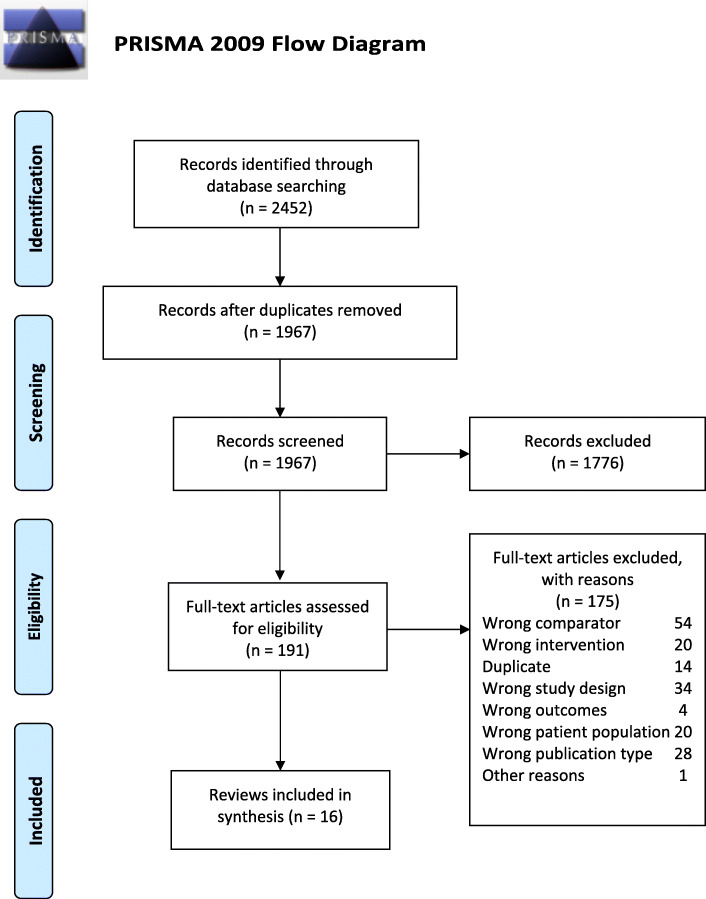
PRISMA flow diagram

Our initial searches yielded 2452 results, and 191 were evaluated as full-text articles following title and abstract screening. According to the eligibility criteria, we included 16 SRs in this overview [15, 23–37]. Figure 2 presents the PRISMA flow diagram. Reasons for exclusion and references to 175 final excluded articles are given in Additional file 4.

Table 1 and Additional file 5 summarise the general characteristics of included SRs. Of the included reviews, two were Cochrane SRs [15, 29], and four were network meta-analyses [28, 31, 32, 34]. Included SRs were published between 2014 and 2020, nine were performed in high-income countries, and seven of them in China. All SRs included meta-analyses except for Harvey 2017 [28]. Three SRs exclusively addressed patients with gastric cancer [23, 25, 30], nine reviews included only patients with gastric cancer including the gastroesophageal junction (GEJ) [26–29, 31–33, 35, 37] and the remaining four considered both patients with esophageal and GEJ cancer [15, 24, 34, 36]. The retrieved SRs included a total of 19 primary studies relevant to our question (See Additional file 6).

**Table 1:** General characteristics of included systematic reviews

Author, year	Country	Time frame	Search strategy/ Database	RCTs (n)	RCTs (n) included in our overview.	P	I	C	O	Funding	Conflicts of interest.	AMSTAR assessment
**Iacovelli 2014** [23]	USA	From January 2004 to February 2014	Cochrane Central Register of Controlled Trials, MEDLINE, PubMed.	5	5	Gastric cancer	CT, Biological, or targeted therapy	BSC / Supportive care	Functional status, OS	No	None	CRITICALLY LOW
**TerVeer 2016** [24]	Netherlands	Up to January 2016	Cochrane Central Register of Controlled Trials, EMBASE, MEDLINE.	28	8	Esophageal, gastric, and GEJ cancer	CT, Biological, or targeted therapy	BSC / Placebo	OS, PFS, Toxicity	No	Yes	CRITICALLY LOW
**Wang 2016** [25]	China	Up to December 31, 2015	Cochrane Library, EMBASE, PubMed	10	3	Gastric cancer	Biological or targeted therapy	Placebo	OS	Public	None	LOW
**Chan a 2017** [26]	Australia	Up to December 2014	Cochrane Central Register of Controlled Trials, EMBASE, PubMed.	15	4	Gastric and GEJ cancer	Biological or targeted therapy	Placebo	OS, PFS, Toxicity, QoL	No	Yes	CRITICALLY LOW
**Chan b 2017** [27]	China	Up to 2016	CINAHL, Cochrane Central Register of Controlled Trials, EMBASE, MEDLINE	5	5	Gastric and GEJ cancer	CT, Biological, or targeted therapy	BSC / Placebo	OS, PFS, Toxicity	No	None	HIGH
**Harvey 2017** [28]	UK	Between 1990 and 2015	PubMed, Scopus.	5	5	Gastric and GEJ cancer	CT	BSC	OS	NR	NR	CRITICALLY LOW
**Janmaat 2017** [15]	Netherlands	Up to 13 May 2015	Cochrane Central Register of Controlled Trials, Clinicaltrials.gov, EMBASE, Google Scholar, MEDLINE, PubMed, Web of Science, WHO International Clinical Trials Registry Platform (ICTRP)	41	5	Esophageal and GEJ cancer	CT, Biological, or targeted therapy	BSC / Placebo / Non-specified	OS	Public	None	HIGH
**Wagner 2017** [29]	Switzerland	Up to June 2016	Cochrane Central Register of Controlled Trials, MEDLINE, Hand searched reference lists from studies, abstracts, conference.	64	3	Gastric and GEJ cancer	CT	BSC	OS	Public	Yes	HIGH
**Wang 2017** [30]	China	Up to December 2015	Embase, Medline, the Cochrane Central Register of Controlled Trials, Cochrane Database of Systematic Reviews, EMBASE, MEDLINE	9	2	Gastric cancer	Biological or targeted therapy	Placebo	Toxicity	NR	None	MODERATE
**Xie 2017** [31]	China	Between January 1st, 2000 and October 1st, 2016	Cochrane Library and Scopus, EMBASE,	23	2	Gastric and GEJ cancer	CT, Biological, or targeted therapy	Placebo	OS	NR	None	CRITICALLY LOW
**Zhu 2017** [32]	Canada	Up to June 2014	American Society of Clinical Oncology abstracts, Cochrane Central Register of Controlled Trials, EMBASE, MEDLINE.	8	5	Gastric and GEJ cancer	CT, Biological, or targeted therapy	BSC / Placebo	OS	Private	Yes	CRITICALLY LOW
**Liu 2018** [33]	China	Up to March 15, 2017	Cochrane Central Register of Controlled Trials, Clinicaltrials.gov, EMBASE, EU Clinical Trials Register, Japan Pharmaceutical Information Center, PubMed.	8	4	Gastric and GEJ cancer	Biological or targeted therapy	Placebo	OS, PFS, Toxicity	Public / Private	None	LOW
**Zhao 2018** [34]	China	Between 2002 and 2017	Cochrane Library, EMBASE, PubMed.	16	6	Esophageal, gastric and GEJ	Biological or targeted therapy	Placebo	OS, PFS, Toxicity	Public	None	CRITICALLY LOW
**Chen 2019** [35]	China	Up to September 2018	PubMed	9	2	Gastric and GEJ cancer	Immunotherapy	BSC / Placebo	OS, PFS, Toxicity	Public	None	CRITICALLY LOW
**van Kleef 2019** [36]	Netherlands	Up to April 2018	Cochrane Central Register of Controlled Trials, EMBASE, MEDLINE.	43	8	Esophageal, gastric and GEJ	CT, Biological, or targeted therapy	BSC / Placebo	QoL	Public	Yes	CRITICALLY LOW
**Wallis 2019** [37]	Canada	Up to October 2, 2018	EMBASE, MEDLINE, PubMed, Scopus.	23	1	Gastric and GEJ cancer	Immunotherapy	Placebo	OS	Private	Yes	CRITICALLY LOW

*GEJ* gastroesophageal junction, *P* patients, *I* intervention, *C* comparator, *O* outcomes, *CT* chemotherapy, *BSC* best supportive care, *OS* overall survival, *PFS* progression-free survival, *QoL* quality of life, *NR* not reported

**Fig. 3:**
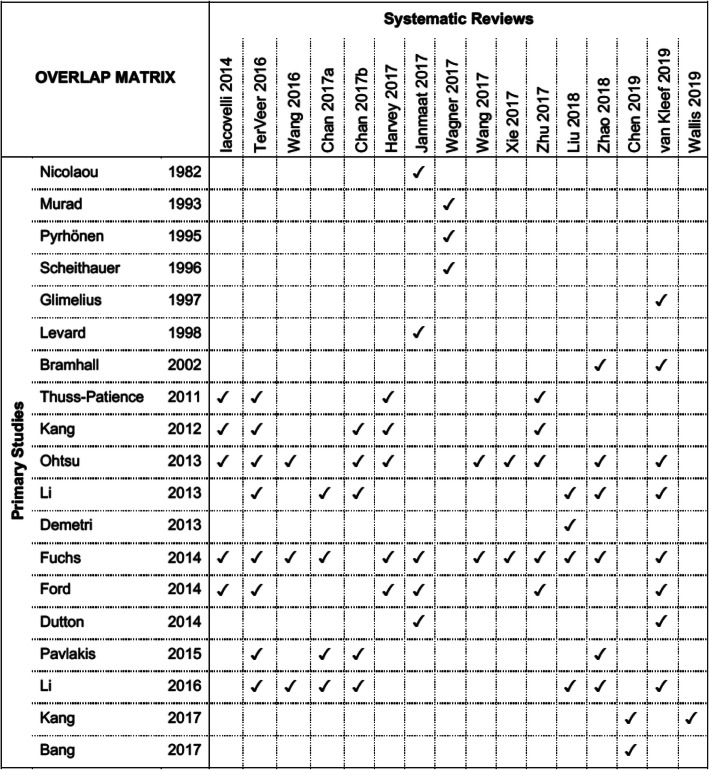
Overlap matrix

Figure 3 shows the overlap matrix of included reviews. The overall CCA was 17.19%, which is considered a very high overlap. Eight primary studies were included in two SRs [24, 36], and five in another five SRs [15, 23, 27, 28, 32].

### Outcomes reported

All reviews pre-specified outcome measures and reported OS, PFS, FS, toxicity, and QoL. None of the reviews examined symptoms related to the disease, admissions, or quality of death.

### Quality assessment of the included systematic reviews

Using the AMSTAR-2 tool, we rated 13 out of 16 SRs (81%) as critically low methodological quality (See Table 2). Only the review of Chan et al. 2017 [27] was evaluated as high quality. Common critical flaws were the lack of report of an explicit protocol for conducting the SR, the lack of information on the sources of funding, and an inadequate assessment of the impact of the risk of bias of primary studies.

**Table 2:** AMSTAR-2 Systematic Reviews

	**Iacovelli 2014** **[**23**]**	**TerVeer 2016** **[**24**]**	**Wang 2016** **[**25**]**	**Chan 2017a** **[**26**]**	**Chan 2017b** **[**27**]**	**Harvey 2017** **[**28**]**	**Janmaat 2017** **[**15**]**	**Wagner 2017** **[**29**]**	**Wang 2017** **[**30**]**	**Xie 2017** **[**31**]**	**Zhu 2017** **[**32**]**	**Liu 2018** **[**33**]**	**Zhao 2018** **[**34**]**	**Chen 2019** **[**35**]**	**van Kleef 2019** **[**36**]**	**Wallis 2019** **[**37**]**
1.	Yes	Yes	Yes	Yes	Yes	No	Yes	Yes	Yes	No	Yes	Yes	No	Yes	Yes	Yes
2.	No	No	No	No	Yes	No	Yes	Yes	Yes	No	No	Yes	No	No	No	No
3.	Yes	Yes	Yes	No	Yes	Yes	Yes	Yes	Yes	No	Yes	Yes	No	No	No	No
4.	Partial Yes	Yes	Yes	Yes	Yes	Partial Yes	Yes	Yes	Yes	Partial Yes	No	Partial Yes	Partial Yes	Partial Yes	Partial Yes	Partial Yes
5.	Yes	Yes	Yes	Yes	Yes	No	Yes	Yes	No	No	Yes	Yes	Yes	Yes	Yes	Yes
6.	Yes	Yes	Yes	Yes	Yes	Yes	Yes	Yes	Yes	Yes	Yes	Yes	Yes	Yes	No	Yes
7.	No	No	Yes	No	Yes	No	Yes	Yes	Yes	No	Yes	No	No	No	No	No
8.	Yes	Partial Yes	Yes	Partial Yes	Yes	Partial Yes	Yes	Yes	Yes	No	Yes	Partial Yes	No	Partial Yes	Partial Yes	Partial Yes
9a.	Partial Yes	Yes	Yes	Yes	Yes	Partial Yes	Yes	Yes	Partial Yes	No	Yes	Yes	Yes	Yes	Yes	Yes
9b.	Includes only RCTs	Includes only RCTs	Includes only RCTs	Includes only RCTs	Includes only RCTs	Includes only RCTs	Includes only RCTs	Includes only RCTs	Includes only RCTs	Includes only RCTs	Yes	Includes only RCTs	Includes only RCTs	No	Includes only RCTs	Includes only RCTs
10.	No	No	No	No	No	No	No	No	No	No	No	No	No	No	No	Yes
11a.	Yes	Yes	Yes	Yes	Yes	No meta-analysis conducted	Yes	Yes	Yes	No	Yes	Yes	Yes	Yes	Yes	No
11b.	Yes	No meta-analysis conducted	No meta-analysis conducted	No meta-analysis conducted	No meta-analysis conducted	Yes	No meta-analysis conducted	No meta-analysis conducted	No meta-analysis conducted	No meta-analysis conducted	Yes	No meta-analysis conducted	No meta-analysis conducted	No meta-analysis conducted	No meta-analysis conducted	No meta-analysis conducted
12.	No	No	Yes	Yes	Yes	No	Yes	Yes	Yes	Yes	Yes	Yes	No	No	No	Yes
13.	No	No	Yes	Yes	Yes	Yes	Yes	Yes	Yes	No	Yes	Yes	No	No	No	Yes
14.	No	Yes	Yes	Yes	Yes	Yes	Yes	Yes	Yes	No	Yes	Yes	No	No	No	No
15.	Yes	No	Yes	Yes	Yes	No	Yes	Yes	Yes	No	Yes	Yes	Yes	Yes	No	No
16.	No	Yes	Yes	Yes	Yes	No	Yes	Yes	No	Yes	Yes	No	Yes	Yes	Yes	Yes
**QUALITY OF THE REVIEW**	CRITICALLY LOW	CRITICALLY LOW	CRITICALLY LOW	CRITICALLY LOW	HIGH	CRITICALLY LOW	LOW	LOW	CRITICALLY LOW	CRITICALLY LOW	CRITICALLY LOW	CRITICALLY LOW	CRITICALLY LOW	CRITICALLY LOW	CRITICALLY LOW	CRITICALLY LOW
Number of critical flaws	6	5	2	3	0	6	1	1	2	8	2	3	5	5	6	6
Number of non-critical flaws	4	3	1	3	1	6	1	1	3	6	1	3	6	5	6	3

1. Did the research questions and inclusion criteria for the review include the components of PICO? 2. Did the report of the review contain an explicit statement that the review methods were established prior to the conduct of the review and did the report justify any significant deviations from the protocol? 3. Did the review authors explain their selection of the study designs for inclusion in the review? 4. Did the review authors use a comprehensive literature search strategy? 5. Did the review authors perform study selection in duplicate? 6. Did the review authors perform data extraction in duplicate? 7. Did the review authors provide a list of excluded studies and justify the exclusions? 8. Did the review authors describe the included studies in adequate detail? 9a. RCT: Did the review authors use a satisfactory technique for assessing the risk of bias (RoB) in individual studies that were included in the review? 9b. NSRI: Did the review authors use a satisfactory technique for assessing the risk of bias (RoB) in individual studies that were included in the review? 10. Did the review authors report on the sources of funding for the studies included in the review? 11a. RCT: If meta-analysis was performed did the review authors use appropriate methods for statistical combination of results? 11b. NSRI: If meta-analysis was performed did the review authors use appropriate methods for statistical combination of results? 12. If meta-analysis was performed, did the review authors assess the potential impact of RoB in individual studies on the results of the meta-analysis or other evidence synthesis? 13. Did the review authors account for RoB in individual studies when interpreting/ discussing the results of the review? 14. Did the review authors provide a satisfactory explanation for, and discussion of, any heterogeneity observed in the results of the review? 15. If they performed quantitative synthesis did the review authors carry out an adequate investigation of publication bias (small study bias) and discuss its likely impact on the results of the review? 16. Did the review authors report any potential sources of conflict of interest, including any funding they received for conducting the review?

### Risk of bias from the primary RCTs included in SRs

**Fig. 4:**
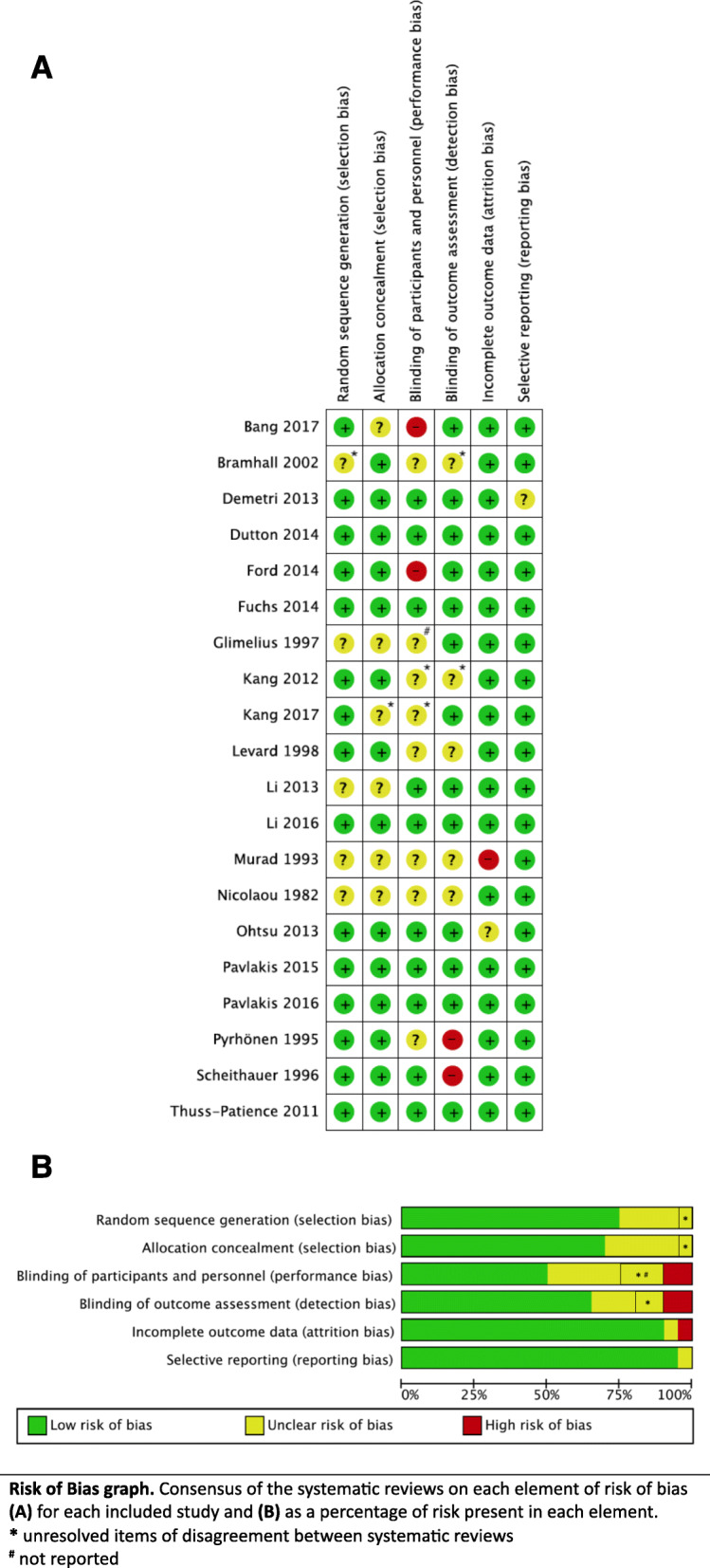
Risk of bias Assessment

Figure 4 summarises the risk of bias of the included primary studies, as reported by the corresponding SR. Most reviews reported the risk of bias using the Cochrane risk of bias tool, while four used the Jadad Scale [23, 25, 30, 31]. Three discrepancies between SR’s assessments remained as “no agreement.” (Bramhall 2002, Kang 2012, Kang 2017). A single domain (performance bias) of one primary trial (Glimelius 1997) was not reported.

### Effectiveness of systemic oncological treatment

**Fig. 5:**
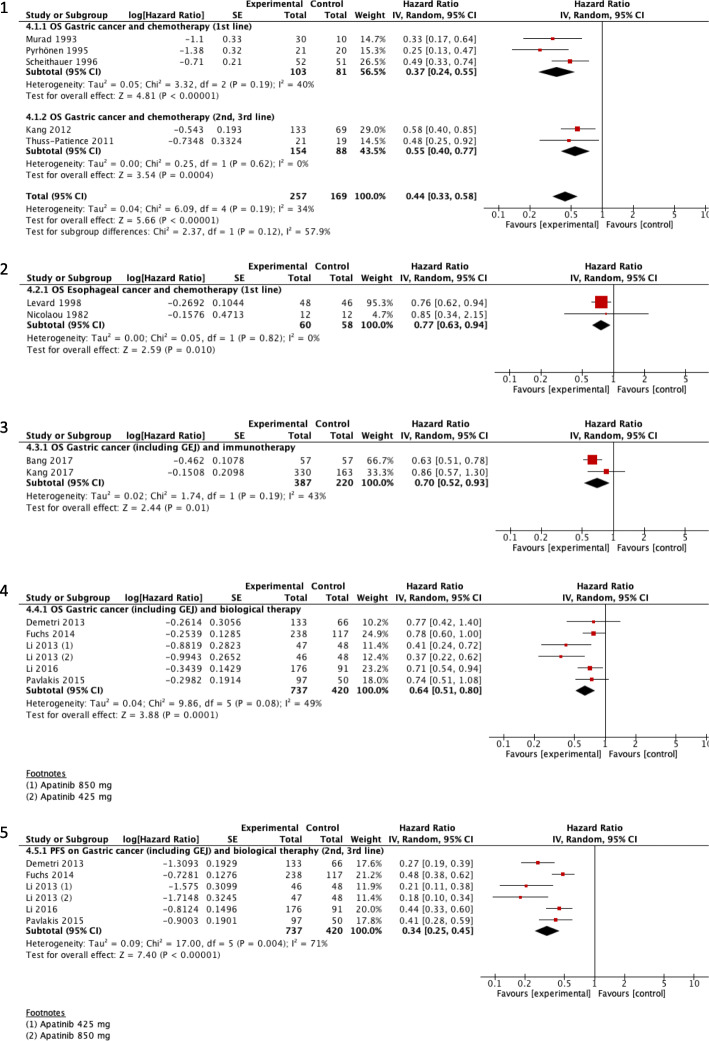
Overall survival and progression-free survival for systemic oncological treatment versus supportive treatment in advanced esophageal or gastric cancers

Due to the variability among the reviews and the outcomes reported, we could combine results only for OS and PFS (Fig. 5).

#### Overall survival

*CT for advanced gastric cancer:* According to our de novo meta-analysis (Fig. 5.1), CT may improve OS over more conservative approaches (HR 0.44, 95%CI 0.33 to 0.58; five studies; low certainty). Wagner 2017 concludes that CT (first-line) improves survival (6.7 months) in comparison to BSC alone. Adding docetaxel to platinum-fluoropyrimidine-based CT regimens may extend OS (just 1 month) with increased toxicity. It is not clear yet whether the benefit of adding a third drug (docetaxel or epirubicin) to a two-drug platinum-fluoropyrimidine CT combination outweighs its toxicity. Consideration of the profile of side effects and the impact of these side effects on the person’s QoL, as well as the tumor burden and necessity to obtain a response rapidly is therefore essential in the choice of the regimen. As a second-line treatment, Iacovelli 2014 reported that CT was able to decrease the risk of death by 27%. In patients with ECOG = 0, a greater benefit was found for chemotherapy with a reduction of the risk of death by 43%. This analysis reports that active and available therapies can prolong survival in patients with advanced gastric cancer with a different outcome based on the initial patient’s performance status.

*CT for advanced esophageal cancer:* According to our de novo meta-analysis (Fig. 5.2), it is very uncertain whether CT improves OS over more conservative approaches (HR 0.77, 95% CI 0.63 to 0.94; two studies; very low certainty). Based on Janmaat’s 2017 analysis, CT can be considered standard care for esophageal cancer. Nevertheless, the main analysis included CT or targeted therapy agent(s) plus control intervention versus control intervention alone.

*Immunotherapy for gastric cancer (including GEJ):* According to our de novo meta-analysis (Fig. 5.3), it is very uncertain whether immunotherapy improves OS over more conservative approaches (HR 0.70, 95% CI 0.52 to 0.93; two studies; very low certainty). A network meta-analysis performed by Zhao 2018 concluded that apatinib, regorafenib, and rilotumumab improved patient OS.

*Biological therapy for gastric cancer (including GEJ):* According to our de novo meta-analysis (Fig. 5.4), biological therapy probably improves OS over more conservative approaches (HR 0.64, 95% CI 0.51 to 0.8; five studies; moderate certainty). Liu 2018 concluded that vascular endothelial growth factor receptor (VEGFR) drugs were effective targeted therapy in advanced or metastatic gastric cancer, and their toxicity is within a controllable range. VEGFR antibody drugs were more effective than VEGFR tyrosine kinase inhibitor drugs in terms of the OS of gastric cancer patients with little toxicity.

*Targeted therapy for esophageal (including GEJ): *According to Dutton et al. (2014), gefitinib (2nd-line) did not improve OS over more conservative approaches (HR 0.90, 95% CI 0.74 to 1.09). Dutton et al. 2014 investigated gefitinib in participants with progression after CT and excluded participants receiving cytotoxic CT, immunotherapy, hormonal therapy, or radiotherapy to the site of measurable or evaluable disease within the 4 weeks before inclusion.

*Biological therapy for gastric cancer:* According to Ohtsu et al. (2013), everolimus 10 mg/d (2nd-line) did not improve OS over placebo (HR 0.90, 95% CI 0.74 to 1.09).

*Targeted therapy for gastric cancer (including GEJ):* According to Bramhalll 2002 there was a modest difference in survival in the intention-to-treat population in favor of marimastat (*P* = 0.07 log-rank test, HR 1.23, 95% CI ^confidence interval^ 0.98 to 1.55). This survival benefit was maintained over a further 2 years of follow-up (*P* = 0.024, HR 1.27, 95% CI 1.03 to 1.57). The median survival was 138 days for placebo and 160 days for marimastat, with a two-year survival of 3 and 9% respectively. A significant survival benefit was identified at study completion in the predefined subgroup of 123 patients who had received prior CT (*P* = 0.045, HR 1.53 (1.00–2.34)). This benefit increased with 2 years of additional follow-up (*P* = 0.006, HR 1.68, 95% CI 1.16 to 2.44).

*CT for esophageal and gastric cancers (including GEJ):* According to Ford et al. (2014), docetaxel as second-line therapy improved OS over BSC (HR 0.67, 95% CI 0.49 to 0.92).

#### Progression-free survival

*Biological therapy for gastric cancers (including GEJ):* According to our de novo meta-analysis (Fig. 5.5), biological therapy (2nd and 3rd line) improved PFS over more conservative approaches (HR 0.34, 95% CI 0.25 to 0.45; I2 71%; five studies). Liu 2018 concluded that VEGFR drugs were effective targeted therapy in advanced or metastatic gastric cancer, and their toxicity is within a controllable range. VEGFR antibody drugs were more effective than VEGFR tyrosine kinase inhibitor drugs in terms of the PFS of gastric cancer patients with little toxicity.

*Biological and targeted therapy for gastric cancer: *According to Bramhall et al. (2002), marimastat as second-line therapy did not improve PFS over placebo (HR 1.32, 95% CI 1.07 to 1.63).

*Biological therapy for gastric cancer: *According to Othsu et al. (2013), everolimus as the second and third line improved PFS over placebo or BSC (HR 0.66, 95% CI 0.56 to 0.78).

*Immunotherapy for gastric cancer (including GEJ):* According to Kang et al. (2017), nivolumab improved PFS over placebo (HR 0.60, 95% CI 0.49 to 0.75).

*Biological therapy for esophageal cancer (including GEJ):* According to Dutton et al. (2014), gefitinib (2nd line) improved PFS over placebo (HR 0.66, 95% CI 0.66 to 0.97).

Table 3 provides a narrative synthesis as an overview of the other outcomes. All the SRs that reported PFS showed a better PFS with SOTs than control [15, 24, 26, 27, 33, 34], while most of the SRs reporting adverse events showed more adverse events than the intervention groups [24, 26, 27, 29–31, 33, 35]. There is scarce data related to QoL, and none of the included SRs reported findings for the outcomes FS, symptoms related to the disease, admissions, or quality of death. Additional file 7 provides SoF tables for the primary outcomes.

**Table 3:** A narrative synthesis of clinical and patient-centered outcomes

SRs Author, year	Reported outcomes											
	OS	RCTs/ RCTs overview	PFS	RCTs/ RCTs overview	FS	Toxicity	RCTs/ RCTs overview^a^	Symptom related to disease	QoL	RCTs/ RCTs overview	Admissions	QoD
Iacovelli 2014 [23]	✓	5/5	NR	–	NR	NR	–	NR	NR	–	NR	NR
Veer 2016 [24]	✓	8/8	2d-line ramucirumab and 2d- or 3d-line everolimus and regorafenib showed limited PFS gain, ranging from 0.3 to 1.6 months	5/8		Targeted agents, either in monotherapy or combined with CT showed increased toxicity compared to BSC and CT-alone	5/8		NR	–		
Wang 2016 [25]	✓	3/3	NR	–		NR	–		NR	–		
Chan 2017 a [26]	✓	4/4	the addition of AAs was associated with improved PFS: HR 0.68 (95% CI 0.63–07.4, *p* < 000001)	4/4		toxicity > = Grade 3: with OR 139 (95% CI 117–165)	2/4		significant improvement in QoL was found with apatinib, in improving insomnia (*p* = 0002), ramucirumab in delaying time to deterioration of PS > = 2 (*p* = 0002) and improving functional functioning and nausea (HR < 075), bevacizumab in slowing deterioration in pain (*p* = 00068), and endostatin in improving global QoL (*p* < 005)	2/4		
Chan 2017 b [27]	✓	4/5	TLT improved PFS (HR 0.29; 95% CI 0.18–0.45)	3/5		more toxicities occurred in the TLT arms	5/5		The QOL data could not be combined in a meta-analysis because only brief descriptions were reported in their final publications	4/5		
Harvey 2017 [28]	✓	4/5	NR	–		NR	–		NR	–		
Janmaat 2017 [15]	✓	5/5	people who receive more CT or targeted therapeutic agents live with less disease progression than people who receive BSC or less therapy	2/5		NR	–		NR	–		
Wagner 2017 [29]	✓	3/3	NR	–		Because of the different ways of reporting, grade I to IV toxicities can be compared only within, but not between studies. Overall, treatment-associated toxicities were higher in the combination of CT arms, but this was usually not statistically significant	3/3		NR	–		
Wang 2017 [30]	✓	2/2	NR	1/2		the addition of TAs to therapies significantly increased the risk of developing severe AEs (RR: 1.12, 95% CI: 1.02–1.24, *P* = 0.02), but not for FAEs (RR: 0.97, 95% CI: 0.65–1.45, *P* = 0.88)	2/2		NR	–		
Xie 2017 [31]	NR	–	NR	–		Compared with other analyzed treatments, ramucirumab has a higher risk of hematological events during its application. Lapatinib is always combined with severe gastrointestinal events. Trastuzumab is proposed for its high efficacy in improving the survival rate and safety, which is proper for most patients	2/2		NR	–		
Zhu 2017 [32]	✓	5/5	NR	–		NR	–		NR	–		
Liu 2018 [33]	✓	4/4	targeting VEGFR drugs significantly improved PFS [HR 0.50, 95% CI (0.34, 0.66), *P* < 0.001]	4/4		Fewer AESIs were observed in the VEGFR-Ab than the VEGFR-TKI drugs. VEGFR drugs were effective, and their toxicity is within a controllable range	4/4		NR	–		
Zhao 2018 [34]	✓	6/6	apatinib (97.5%) was most likely to improve PFS, followed by regorafenib (86.3%) and rilotumumab (65.4%).	6/6		bevacizumab (85.5%) was likely to get the lowest severe AEs, followed by sunitinib (63%)	5/6		NR	–		
Chen 2019 [35]	✓	2/2	NR	1/2		The most common grade ≥ 3 TRAEs were fatigue, aspartate aminotransferase increased, hepatitis, pneumonitis, colitis, hypopituitarism. The TRAE incidence of anti-PD-1/PD-L1 was less than chemotherapy (TRAE RR = 0.64 *p* < 0.001; ≥3 TRAE RR = 0.37 *p* < 0.001). The incidence of ≥3 TRAEs of anti-PD-1/PD-L1 treatment was less than that of anti-CTLA-4 (11.7% vs 43.9%)	2/2		NR	–		
van Kleef 2019 [36]	NR	–	NR	–		NR	–		taxanes and targeted agents could provide HRQoL benefit beyond the first-line compared with BSC	8/8		
Wallis 2019 [37]	✓	1/4	NR	–		NR	–		NR	–		

*AAs* Anti-angiogenic agents, *BSC* best supportive care, *AE* Adverse events, *CT* chemotherapy, *FAE* Fatal adverse events, *FS* Functional status, *OS* Overall survival, *PFS* Progression-free survival, *QoD* Quality of death, *QoL* Quality of life, *TA* Targeted agents, *TLT* third-line treatment

Outcomes in bold are primary outcomes

OS has been presented in Fig. 4 (meta-analysis)

^a^ RCTs: randomized controlled trials relevant to our question / RCTs overview: total randomized controlled trials included in the SR

## Discussion

This overview provided a comprehensive synthesis of the available evidence regarding the effectiveness of SOT compared with BSC or placebo administered in patients with advanced esophageal or gastric cancers. The current analyses revealed that it is uncertain whether SOT, such as CT, immunotherapy, biological and targeted therapy improve OS and PFS over more conservative approaches due to the very low certainty of evidence. Most reviews had a critically low methodological quality and did not include outcomes considered important in decision-making.

### Context

Over the last two decades, cancer care’s aggressiveness near the EOL has emerged as a growing concern [38, 39]. For instance, the American Society of Clinical Oncology (ASCO) recommends avoiding the use of CT near the EOL due to the absence of evidence supporting its clinical value [40]. This is in line with our results, confirming the limited attention that patient-centered outcomes have received so far. Usually, SRs only meta-analyze clinical outcomes such as OS or PFS but do not include others like FS, toxicity, or symptoms related to the disease, admissions to the hospital, or palliative care provided during the last year. All of these outcomes are particularly relevant for patients with a high risk of dying in the short or medium term. These evidence gaps in reporting essential outcomes for patients could reflect either lack of primary data availability in the respective studies or event or lack of interest by authors of SRs to analyze this data actively.

Treatment for locally advanced, unresectable esophageal or gastric cancers remains highly controversial. For example, the European Society of Medical Oncology (ESMO) guidelines are not well-defined in this regard [41]. They recommend systemic treatment (CT) for patients with inoperable locally advanced and/or metastatic (stage IV) disease, based on improved survival and QoL compared to BSC alone (I, A). However, comorbidities, organ function, and PS must always be taken into consideration. (II, B). This recommendation is based on small or large RCTs with suspicion of bias (lower methodological quality) or meta-analyses of such trials or trials with demonstrated heterogeneity. As we have stated, our overview’s results do not go in the same direction as those guidelines recommendations due to the low quality of the evidence to support an active systemic treatment in patients with advanced stages with a high risk of dying in the short or medium-term. It is important to note that all the mentioned guidelines recommendations for all the different SOT regimens are mainly based on a potential OS improvement of a few months and rarely consider patient-centered outcomes.

Although there is evidence suggesting that SOT’s use near the EOL is not related to its likelihood of providing a benefit [27, 42–44], our analysis could not confirm any significant differences between SOT and BSC for all the outcomes. The discussion about changing the focus of treatment to providing symptomatic and supportive care is complex. Little literature addresses the magnitude of financial, psychological, or physical harms of medication overuse in cancer, even when it could be substantial [45]. Smith and Hillner have proposed for patients with advanced cancer, changes in medical oncologists’ behavior, and changes in their attitudes and practices that will bend the cancer-cost curve [46]. For patients with advanced esophageal and gastric cancers, we can consider the following aspects: a) to limit second-line and third-line treatment for metastatic cancer to sequential monotherapies; b) to limit CT to patients with good PS; c) to limit further CT to clinical trials in the case of patients who are not responding to three consecutive regimens. Besides, regarding changes in attitudes and practice, we agree with the author that better integration of palliative care into usual oncology care must be discussed.

One problem to solve is how supportive care and BSC are implemented in RCTs when used as a comparison treatment arm. Reviews of the cancer clinical trial literature found that RCTs poorly define and standardize BSC as a clinical trial control arm [47, 48]. Such studies risk systematically over-estimating the net clinical effect of the comparator arms. The vast majority of the studies did not meet the WHO guidelines on BSC because palliative care therapies were not recommended or integrated into care.

Another issue is how studies entitle the patients reported outcomes (PROs). The heterogeneity in the constructs, measures and analytic is very challenging to interpret [49]. As we found in our study, especially in adverse events, it is important to take responsibility for the need to strengthen the rigor of PROs in cancer trials or studies reports. It is also essential to acknowledge the discordances between patient and clinician reports regard the symptoms and severity [50, 51]. It could be useful to follow the Patient-Reported Outcome Terminology Criteria for Adverse Events (PRO-CTAE) tools in case to be necessary and finally to consider all the submitted PROs as important supportive data improving the validity, reliability, and precision of adverse events report.

### Limitations

We are aware that our research may have limitations. Firstly, the main limitation of this overview arises from heterogeneity amongst active treatments assessed. In this regard, we assessed the included studies’ heterogeneity and undertook analysis by type of SOT.

Secondly, we found that the overlapping RCT distribution may over-represent samples from these primary studies. Nevertheless, we reported this overlap and quantified it using the mentioned CCA method to help us consider questions that could affect our overview’s comprehensiveness and complexity.

Finally, the risk of bias assessment of primary studies was not performed directly on the original studies but each SR, resulting in an incomplete assessment for some studies and potentially hindering the overall assessment. We plan to carry out an evidence map and a new SR to address these limitations.

### Implications

This overview did not identify solid evidence for administering SOT over BSC for patients with advanced esophageal or gastric cancers. Involved doctors and patients should be aware of the limited benefits that intensive SOT can provide when the disease is very advanced. The therapeutic decisions for patients with advanced esophageal or gastric cancers must consider their FS, values and preferences, and potential side effects of treatments. However, to enable patients to make informed choices, they should be provided with balanced information. Unfortunately, as shown in our overview, clinical trials and SRs barely report patient-centered outcomes.

It is important to note that almost all RCTs for patients with esophageal or gastric cancers currently focus on treating the disease’s early stages. However, the evidence collected in this overview shows that it is still necessary to evaluate how to treat patients in advanced stages. We claim future clinical trials and reviews to address SOT’s impact in patients with advanced stages at high risk of dying in the short or medium term. We do so by considering that before comparing intensive treatments, these should demonstrate their advantages over more conservative approaches such as BSC, not only on survival but also on patient-centered outcomes. High-quality SRs with complete reporting of design, methodology, and analysis of results could perform pre-planned subgroup analyses to identify those groups of patients more prone to benefit from intensive systemic treatments and avoid the accompanying side effects.

## Conclusions

This overview suggests that there is a large uncertainty on the effectiveness of SOT for advanced esophageal or gastric cancers that could provide a complete understanding of benefits and side effects. Broader research, including high-quality SRs and further RCTs that consider a thorough assessment of patient-centered outcomes, is needed to identify improvement targets to optimize cancer care value.

## Supplementary Information

**Additional file 1.** Protocol study.

**Additional file 2.** Complete PRISMA checklist. The PRISMA checklist was completed in full with the page number of the paper which reports the information that meets the criteria of the checklist.

**Additional file 3.** Search strategy.

**Additional file 4.** List of excluded reviews and justification for the exclusions.

**Additional file 5.** Further characteristics of included systematic reviews.

**Additional file 6.** References RCTs.

**Additional file 7.** GRADE.

## Data Availability

The protocol of the current study is available in the OSF repository, https://osf.io/7chx6/ Accessed 11 January 2021 (DOI 10.17605/OSF.IO/7CHX6). Search strategies needed to replicate the study are included in the supplement materials file.

## Abbreviations

AMSTAR: A MeaSurement Tool to Assess systematic Reviews
ASCO: American Society of Clinical Oncology
BSC: Best supportive care
CCA: Corrected covered area
CI: Confidence interval
CT: Chemotherapy
EMBASE: Excerpta Medica dataBASE
EOL: End-of-life
ESMO: European Society for Medical Oncology
FS: Functional status
GEJ: Gastro-esophagic junction
GRADE: Grading of Recommendations Assessment, Development, and Evaluation
HR: Hazard ratio
MEDLINE: Medical Literature Analysis and Retrieval System Online
OR: Odds ratio
OS: Overall survival
PFS: Progression- free survival
PRISMA: Preferred Reporting Items for Systematic reviews and Meta-Analyses
PROSPERO: International Prospective Register of Systematic Reviews
PROs: Patients reported outcomes
PRO-CTAE: Patient-Reported Outcome Terminology Criteria for Adverse Events
QoL: Quality of life
RCTs: Randomised controlled trials
SoF: Summary of finding
SRs: Systematic reviews
SOT: Systemic oncological treatment
VEGFR: Vascular endothelial growth factor receptor
WHO: World Health Organization
