# Supplementary material for: Efficacy of systemic oncological treatments in patients with advanced esophageal or gastric cancers at high risk of dying in the middle and short term: an overview of systematic reviews
Source: BMC Cancer. 2021 Jun 16;21:712. doi: 10.1186/s12885-021-08330-5 (PMC8207789; doi:10.1186/s12885-021-08330-5)
Supplement: Supplementary file 1 — Additional file 1. Protocol study. [file 12885_2021_8330_MOESM1_ESM.pdf]

Efficacy of systemic oncological treatments in patients with advanced esophageal and gastric cancers at high risk of dying in the middle and short term: Protocol for an overview of systematic reviews

Authors:

Santero M (1), Pérez-Bracchiglione J (2), Acosta-Dighero R (4), Antequera Martín A(1), Auladell-Rispau A (1), Dorantes-Romadía R (1), Lara-Vinueza A (1), Quintana M J (1), Requeijo C (1), Rodríguez-Grijalva G (1), Salas-Gama K (1), Salazar-Núñez J (1) ,Solà I (1,3), Urrútia G (1,3), Bonfill-Cosp X (1,3)

(1) Iberoamerican Cochrane Centre, Biomedical Research Institute Sant Pau (IIB Sant Pau), Barcelona, Spain
(2) Interdisciplinary Centre for Health Studies (CIESAL), Universidad de Valparaíso, Viña del Mar, Chile
(3) CIBER Epidemiología y Salud Pública (CIBERESP), Barcelona, Spain
(4) Department of Physical Therapy, Faculty of Medicine, University of Chile, Santiago, Chile

This study is funded through a grant from Instituto de Salud Carlos III (PI18/00034) co-financed by funds from the European Regional Development Fund

Corresponding author:

Marilina Santero, C/ Sant Antoni Maria Claret, 167, Pavelló 18, planta 0. 08025. Barcelona, España. Teléfono: +34 93 553 78 14 Fax: +34 93 553 78 09. MSantero@santpau.cat

ABSTRACT

Background: Esophageal and gastric cancers are an important public health problem worldwide, with most patients presenting with advanced-stage disease and consequently with a poor prognosis. Systemic oncological treatments have been widely used over more conservative approaches, such as supportive care. Nevertheless, the effectiveness in this scenario is not sufficiently clear.

Objectives: To make a comprehensive synthesis of the available evidence regarding the effectiveness of systemic oncological treatments, and to compare them with the best supportive care or placebo administered in patients with advanced esophageal and gastric cancers in an end-of-life context.

Methods: This is a protocol for a systematic overview of reviews. We will search five databases: PubMed, EMBASE, The Cochrane Library, Epistemonikos and PROSPERO. We will consider systematic reviews of randomized controlled trials in adults diagnosed with advanced esophageal or gastric cancers in an end-of-life context, evaluating the effect of any systemic oncological treatment, and any supportive care or placebo as comparison. Primary outcomes will be survival, quality of life, functional status and toxicity. Two authors will independently screen articles for inclusion using *a priori* criteria. One author will assess the quality of included SRs and extract data, while another reviewer will cross-check this process. We will assess overlapping primary studies using the corrected covered area formula. Presentation of results will align with guidelines of the Cochrane Handbook of Systematic Reviews. If possible, we will perform a *de novo* meta-analysis with the data reported for each primary study in systematic reviews. We will assess the certainty of evidence using the GRADE approach.

Expected results: Our overview will synthesize the broad degree of information available and could be used by healthcare managers, administrators and policymakers to guide resource allocation decisions and inform local implementation and optimization of treatments in patients with advanced esophageal and gastric cancers in an end-of-life context.

Keywords: Esophageal Cancer, Gastric Cancer, Antineoplastic Agents, Biological Therapy, Molecular Targeted Therapy, Immunotherapy, Review Literature as Topic.

INTRODUCTION

Worldwide, esophageal and gastric cancers are an important public health problem, with approximately 509,000 and 783,000 deaths in 2018, respectively.(1) With combined mortality of over 1.2 million they are the second most common cause of cancer death after lung cancer. These cancers are multifactorial diseases and different risk factors have been associated with their development such as genetics, male gender, lifestyle, nutrition, alcohol, smoking, infection, and *H.pylori* bacteria. (2)(3) Even reports have shown a decrease in mortality rates for gastric cancer over the past 20 years, higher steady mortality rates for esophageal cancer are present in mainly Western Pacific and European regions.(4) (Figure 1 and Figure 2)

Figure 1: Mortality rates for gastric cancer between 1990 and 2017.

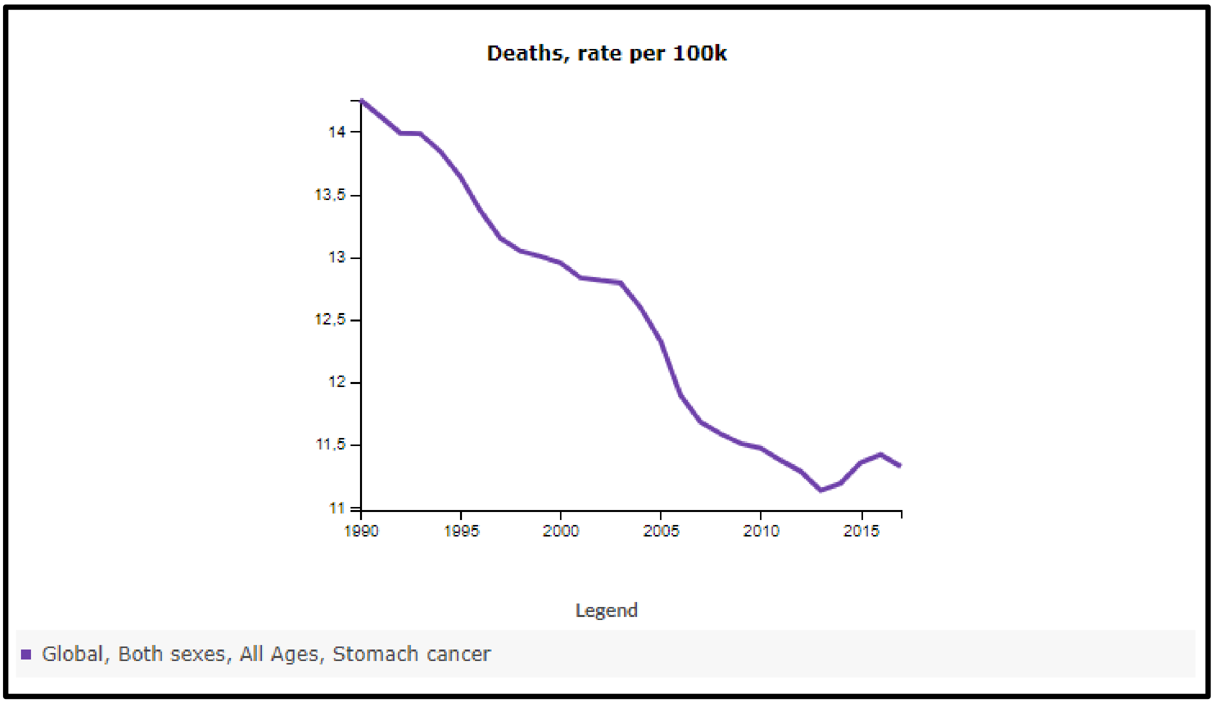

Source: Institute for Health Metrics and Evaluation

Figure 2: Mortality rates for esophageal cancer between 1990 and 2017, by WHO regions.

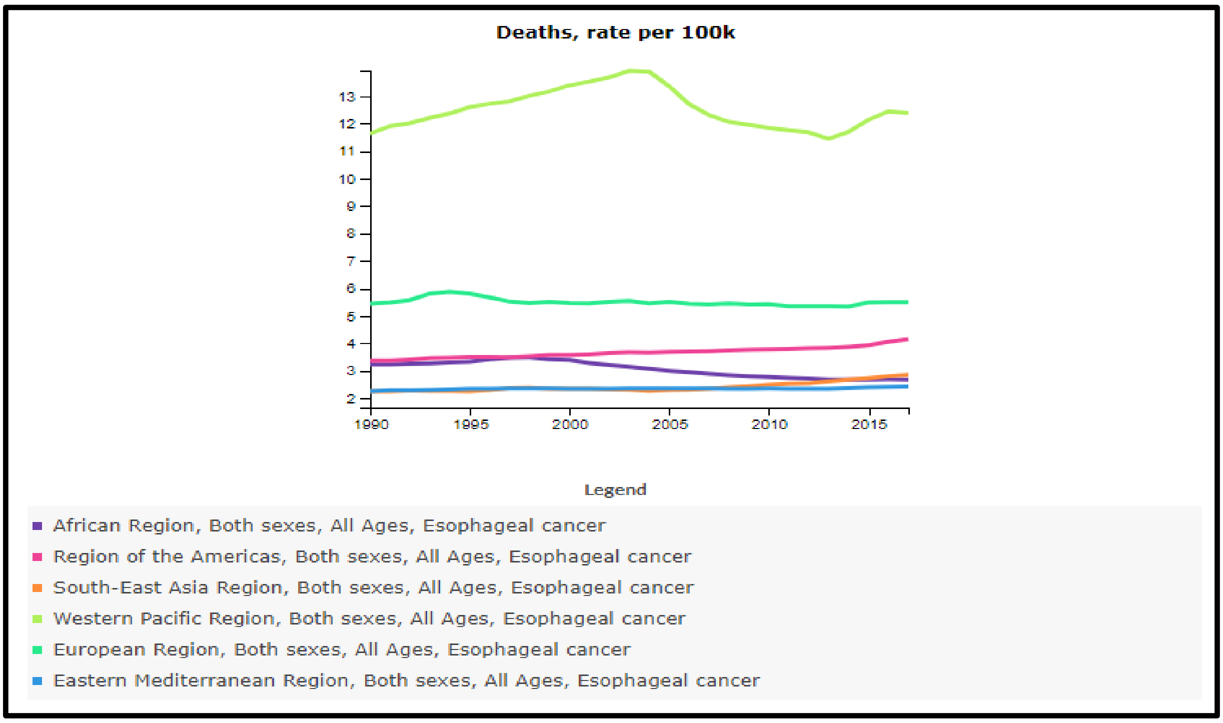

Source: Institute for Health Metrics and Evaluation

Despite its relatively low incidence, both cancers are extremely aggressive and often have a poor prognosis since the diagnosis is usually late.(5) In a distant stage, esophageal and gastric cancers have less than 30% survival at 1 year, and less than 5% at 5 years. (Table 1)

Table 1: Survival rates at 1, 3 and 5 years for esophageal and gastric cancers, stratified by stage at diagnosis *(6)*

	**Localised**			**Regional**			**Distant**		
	1y	***3y***	***5y***	***1y***	***3y***	***5y***	***1y***	***3y***	***5y***
**esophageal**	67.0	47.6	40.0	59.2	29.6	21.8	**27.4**	**6.7**	**4.2**
**gastric**	80.9	69.8	65.0	66.2	36.7	28.8	**25.4**	**7.0**	**4.5**

Due to the above, patients are at high risk of dying in the middle and short term, which has been conceptualized as a time frame called “end of life” (EOL).(7) The definition of this specific concept varies among authors, but overall it can be described as a period that precedes the natural death of an individual from a progressive pathological process unlikely to be stopped by medical care.(8)

The use of systemic treatments has been a major priority for esophageal and gastric cancers, with studies testing chemotherapy, targeted therapy and immunotherapy to improve survival and quality of life.(9,10) However, its use in the EOL context is subject to evaluation mostly because of concerns due to its effectiveness and the impact it has on the quality of life (QOL). Some authors reported patients experience emotional distress, a severely reduced QOL and a range of diagnosis-specific and treatment-related problems and side effects such as difficulties with nutrition or elimination.(11) The overuse of systemic treatments close to death could be an indicator of poor quality medical care, defined as the underuse of practices of known effectiveness, or the use of practices of equivocal effectiveness according to provider rather than patient’s preferences.(12) Among patients with esophageal and gastric cancers, short survival time is associated with several indicators of low quality EOL care, suggesting that a proactive palliative care approach is imperative.(13)

Best supportive care (BSC), as palliative care, includes a group of interventions of a multidisciplinary approach, such as symptomatic control by radiotherapy (other than

primary site), palliative surgery, management of antineoplastic-treatment-related toxicities, analgesia and psychological or social assistance.(14)(15) The American College of Surgeons has recommended that palliative care should be integrated early into the course of the disease, concurrently with active treatment.(16) However, treatment with surgery (17) or chemotherapy (18) has been associated with underuse of palliative care in terms of palliative care consultation (18,19) and late hospice referral (20), which indicates that treatment characteristics may influence the quality of EOL care.

Since the majority of patients with esophageal and gastric cancers are likely to die in the middle or short term, it is of central importance to evaluate the appropriateness of the systemic treatments compared to the existing alternatives, such as BSC, in terms of effectiveness, while having special consideration for patient's QOL near death and relief of the significant physical and psychological symptomatic burden that these patients present. Synthesizing and combining relevant data from existing systematic reviews or meta-analyses to make better decisions is required.

Thus, this study aims to make a comprehensive synthesis of the available evidence regarding the effectiveness of systemic oncological treatments, and to compare them with BSC or placebo in patients with advanced esophageal and gastric cancers in an EOL context.

METHODS

Design

The present article is part of a wider protocol for an overview of systematic reviews for patients with advanced non-intestinal digestive cancer at high risk of dying in the middle and short term, (21) and will respond explicitly to patients with advanced esophageal and gastric cancers in an EOL context. Although there is limited methodological guidance to conduct overviews, we will conduct the study according to rigorous standards aligned with Cochrane methodology.(22) We will report this protocol adhering to the PRISMA-P reporting guidelines.(23)

Eligibility criteria

We will use the PICOT framework (Patients, Intervention, Comparison, Outcomes, Type of study) to guide our eligibility criteria.(23, 24)

Type of studies

We will include only systematic reviews that assess the impact of systemic oncological treatments in advanced esophageal and gastric cancer patients at high risk of dying in the middle and short term, published from 2008 onwards. We will consider a systematic review any type of secondary research that states: i) explicit eligibility criteria or research questions, ii) a structured search strategy (defined as

explicit search terms and data frame, in at least two databases), iii) explicit inclusion criteria and screening methods, iv) precise assessment of the quality or risk of bias of each included study, and v) explicit approach to data analysis and synthesis.(25) We will exclude any primary research (such as randomized clinical trials, quasi-experimental studies, observational studies, and descriptive studies), clinical practice guidelines, and any non-systematic review (such as narrative reviews).

Type of patients

We will consider eligible reviews including adult patients (over 18 years old), with a diagnosis of esophageal, gastroesophageal junction, or gastric cancer, primary or recurrent, in stage IIIb, IIIc or IV, or described as advanced or metastatic by the authors of the systematic review at the moment of the intervention. We will exclude lymphatic, stromal and neuroendocrine cancers.

Type of interventions

For the intervention arm, studies will be considered eligible if they include any chemotherapy (CT), either monotherapy or in combination, or another systemic oncological treatment (biological, targeted therapy or immunotherapy), whether individual or combined, with or without supportive care. We will exclude reviews that consider as an intervention only surgery or radiotherapy, as well as reviews that consider only CT as adjuvant or neoadjuvant therapies.

We will consider as comparison any supportive treatment used as BSC. (26) Studies that do not explicitly define the intervention of the control group, or studies with placebo as the intervention of the control group, will also be included. We will exclude reviews with a control group that includes any CT, biological therapy, targeted therapy or immunotherapy. We will also exclude comparisons with surgical or radiotherapeutic treatments with non-palliative intent.

Type of outcomes

We will consider a systematic review eligible if it includes any of the following outcomes:

1. Clinical outcomes:

    a. Survival: As a dichotomous outcome (at 3, 6, 9, 12, 24 months) and as a time-to-event outcome.

    b. Progression-free survival: As a dichotomous outcome (at 3, 6, 9, 12, 24 months) and as a time-to-event outcome.

    c. Functional status: Measured with Karnofsky or ECOG scale.

    d. Toxicity: Measured as moderate or severe adverse events, according to standardized classification.

2. Patient-centered outcomes:
    a. Symptoms related to the disease: Measured with validated scales that assess one or more symptoms.
    b. Quality of life: Measured with validated scales.
    c. Admissions to a hospital or long-term center, or emergency consultations: Measured as the total number of admissions and days of admission during the follow-up period.
    d. Quality of death:
        i. Admission to a hospital at the end-of-life: Admission to the hospital in the last 30 days of life.
        ii. Palliative care provided during the last year: As a dichotomous outcome.
        iii. Place of death: Home, institutionalized (health community center or residence), hospitalized (intensive care or other).

We will consider the following as primary outcomes: survival, quality of life, functional status and toxicity.

Search methods for identification of studies

We will search MEDLINE (access via PubMed), EMBASE (access via OVID), the Cochrane Database of Systematic Reviews, and Epistemonikos from inception onwards. We will design search strings adapted to the requirements of each database that will combine controlled vocabulary and search terms related to the main concepts of our clinical question. We will use search filters for systematic reviews. The protocol for an overview of systematic reviews for patients with advanced non-intestinal digestive cancer at high risk of dying in the middle and short term (21) provides the search strategy for PubMed.

We will also search in PROSPERO to identify protocols for eligible reviews. We will ask experts in the field for relevant studies, and we will perform a reverse snowballing process with the included studies. We will not use any other strategy to search for grey literature.

Selection of studies

The review process will be facilitated by using Covidence software (www.covidence.org). Two previously trained reviewers will perform an independent title and abstract screening of the results obtained from the search. A third reviewer will solve any disagreements. Afterwards, two reviewers will conduct the full-text screening, also with a third author solving any disagreement.

Data extraction and Risk of Bias Assessment

One reviewer will extract data from the included studies, using a data extraction sheet that will be previously piloted. A second author will cross-check this process. From the included studies, we will extract both synthesized findings and disaggregated data for each primary study considering the outcomes of interest, as reported by the respective systematic review. We will extract data directly from the primary studies only if the systematic review does not provide it.

One author will assess the risk of bias for each included systematic review using the AMSTAR-2 tool.(27) A second author will cross-check this assessment. We will report the risk of bias assessment of primary studies undertaken by the authors of each systematic review. If two or more systematic reviews have different assessments of the same primary study, we will consider the assessment of the review with the best methodological quality according to the AMSTAR-2 assessment.

Assessment of overlap between primary studies

We will build a matrix of evidence to assess a possible overlap between primary studies within systematic reviews. In this matrix, the columns will represent all the included systematic reviews, and the rows will consider the primary studies included in each review (Figure 2). With this matrix, we will calculate the corrected covered area (CCA). We will consider a CCA below 5% as slight overlap, a CCA ≥5% and <10% as moderate overlap, a CCA ≥10% and <15% as high overlap, and a CCA ≥15% as a very high overlap.(28) We will incorporate these findings at the moment of data extraction and discussion of results.

Figure 2: Matrix of evidence for assessing the overlap between primary studies within systematic reviews.(28)

		Columns			
		SR 1	SR 2	SR 3	...
Rows	Primary study 1	X			
	Primary study 2	X		X	
	Primary study 3	X	X	X	
	Primary study 4		X		
	Primary study 5		X	X	
	...				

Data synthesis and analysis

For each comparison, we will perform a *de novo* meta-analysis based on the data of each primary study extracted from the systematic review. We will analyze dichotomous outcomes with an odds ratio (OR), continuous outcomes with a mean difference or standardized mean difference, and time-to-event outcomes with hazard ratios (HR), all of these with a 95% confidence interval.

We will assess the heterogeneity of the included studies with I2. We will consider an I2<50% as low heterogeneity, I2 >50% and <90% as high, and >90% as very high. If heterogeneity is below 90%, we will perform a meta-analysis using the random-effects model. If heterogeneity is very high, we will only describe the results without performing a meta-analysis.

We also plan to undertake a subgroup analysis according to the methodological design of primary studies (experimental vs. non-experimental) and main subtypes of cancer (SCC vs. AC). We will also conduct a sensitivity analysis, considering only studies in which comparison is described explicitly as BSC, considering that it has been reported that this specific therapeutic intervention could be reported incompletely and inconsistently between studies.(29)

We will assess the presence of possible publication bias by visual inspection of a funnel plot for the 12-month survival comparison. If there are 10 or more included studies in a specific funnel plot, we will also consider using the Egger test.

Assessment of certainty of evidence

We will assess the certainty of the evidence for each primary outcome according to the GRADE guidance.(30) We will make a “Summary of Findings” (SoF) table for the following outcomes: i) survival, ii) symptoms related to the disease, iii) functional status, and iv) quality of life. We will classify the certainty of the evidence for each outcome as high, moderate, low or very low. For outcomes with data from trials, we will initially rate their certainty as high, which will be lowered in the presence of important bias, indirectness or inconsistency in results, imprecision in estimates, or suspicion of publication bias. Evidence from observational studies will initially be rated as low. However, in the absence of limitations as mentioned above, we will consider increasing certainty if the large magnitude of effects or a dose-response gradient are observed, or if possible confounding factors do not have much impact into effect estimates. We will explicitly state if a specific clinical question has no included studies. In this case, we will not assess the certainty of evidence.

We will also report the main findings of the SoF table in simple language, according to their specific assessment of the certainty of evidence. This table will provide key information concerning the quality of evidence, the magnitude of effect of the

interventions examined, and the sum of available data on all important outcomes for a given comparison.

STRENGTHS AND LIMITATIONS

This protocol has several strengths. Our search strategy includes the most extensive databases on the topic. Besides, all our screening process will be done independently by two reviewers. Also, we will assess the methodological quality of each included systematic review, and we will additionally assess the overlap between primary studies both visually and statistically, incorporating this in the data extraction process and the discussion. Last, we intend to perform *de novo* meta-analysis, which will allow the assessment of certainty of evidence using GRADE.

However, some limitations should be noted. Firstly, the risk of bias assessment of the primary studies will not be done directly from the original articles, but from the systematic reviews. This may be relevant if there are two different tools for assessing the risk of bias in the same comparison since it could hinder the assessment of the certainty of evidence. Another possible scenario is that authors of two different systematic reviews with an overlapped primary study could differ in their assessment of the risk of bias. We plan to face this by critically assessing the systematic reviews with the AMSTAR-2 tool, considering, in such cases, the reports given by the review with the best methodological quality. Secondly, since this overview has a broad scope, we expect high or very high heterogeneity for some outcomes. We will assess this using I2, and also planning *a priori* subgroup analyses.

In conclusion, this will be an overview of systematic reviews and meta-analyses of the effectiveness of chemotherapy or other systemic treatments in patients with advanced esophageal and gastric cancers in an EOL context. We will make a comprehensive synthesis of the available evidence which could be used by healthcare managers, administrators and policymakers to guide resource allocation decisions and inform local implementation and optimization of treatments.

REFERENCES

1. Bray F, Ferlay J, Soerjomataram I, Siegel RL, Torre LA, Jemal A. Global cancer statistics 2018: GLOBOCAN estimates of incidence and mortality worldwide for 36 cancers in 185 countries. CA Cancer J Clin. 2018 Nov;68(6):394–424.

2. Rustgi AK, El-Serag HB. Esophageal Carcinoma [Internet]. Vol. 371, New England Journal of Medicine. 2014. p. 2499–509. Available from: http://dx.doi.org/10.1056/nejmra1314530

3. El-Serag HB, Hashmi A, Garcia J, Richardson P, Alsarraj A, Fitzgerald S, et al. Visceral abdominal obesity measured by CT scan is associated with an increased risk of Barrett's oesophagus: a case-control study [Internet]. Vol. 63, Gut. 2014. p. 220.2–229. Available from: http://dx.doi.org/10.1136/gutjnl-2012-304189

4. Website [Internet]. [cited 2020 Mar 10]. Available from: Global Burden of Disease Study (GBD) [updated 2019/8/282019/8/28]. Available from: http://ghdx.healthdata.org/gbd-results-tool.

5. Pennathur A, Gibson MK, Jobe BA, Luketich JD. Oesophageal carcinoma [Internet]. Vol. 381, The Lancet. 2013. p. 400–12. Available from: http://dx.doi.org/10.1016/s0140-6736(12)60643-6

6. Website [Internet]. [cited 2020 Apr 10]. Available from: SEER*Explorer 2019 [updated 2019/4/15 2019/8/222019/8/22]. Available from: https://seer.cancer.gov/explorer/application.php?site=1&data_type=1&graph_type=2&compareBy=sex&chk_sex_3=3&chk_sex_2=2&chk_race_1=1&chk_age_range_1=1&chk_data_type_1=1&advopt_precision=1&advopt_display=2&showDataFor=race_1_and_age_range_1_and_data_type_1.

7. &na;, &NA; Redefining Our Definition of End of Life [Internet]. Vol. 3, Journal of Hospice & Palliative Nursing. 2001. p. 5–6. Available from: http://dx.doi.org/10.1097/00129191-200103010-00001

8. Hui D, Nooruddin Z, Didwaniya N, Dev R, De La Cruz M, Kim SH, et al. Concepts and definitions for "actively dying," "end of life," "terminally ill," "terminal care," and "transition of care": a systematic review. J Pain Symptom Manage. 2014 Jan;47(1):77–89.

9. Wagner AD, Grothe W, Haerting J, Kleber G, Grothey A, Fleig WE. Chemotherapy in advanced gastric cancer: a systematic review and meta-analysis based on aggregate data. J Clin Oncol. 2006 Jun 20;24(18):2903–9.

10. Wagner AD, Syn NLX, Moehler M, Grothe W, Yong WP, Tai B-C, et al. Chemotherapy for advanced gastric cancer [Internet]. Cochrane Database of Systematic Reviews. 2017. Available from: http://dx.doi.org/10.1002/14651858.cd004064.pub4

11. Blazeby JM, Farndon JR, Donovan J, Alderson D. A prospective longitudinal study examining the quality of life of patients with esophageal carcinoma. Cancer. 2000 Apr 15;88(8):1781–7.

12. Bergman J, Laviana A. Opportunities to maximize value with integrated palliative care [Internet]. Journal of Multidisciplinary Healthcare. 2016. p. 219. Available from: http://dx.doi.org/10.2147/jmdh.s90822

13. Dalhammar K, Malmström M, Schelin M, Falkenback D, Kristensson J. The impact of initial treatment strategy and survival time on quality of end-of-life care among patients with oesophageal and gastric cancer: A population-based cohort study. PLoS One. 2020 Jun 22;15(6):e0235045.

14. Cunningham SC, Schulick RD. Palliative management of gastric cancer. Surg Oncol. 2007 Dec;16(4):267–75.

15. Janmaat VT, Steyerberg EW, van der Gaast A, Mathijssen RH, Bruno MJ, Peppelenbosch MP, et al. Palliative chemotherapy and targeted therapies for esophageal and gastroesophageal junction cancer. Cochrane Database Syst Rev. 2017 Nov 28;11:CD004063.

16. Task Force on Surgical Palliative care, Committee on Ethics. Statement of principles of palliative care. Bull Am Coll Surg. 2005 Aug;90(8):34–5.

17. Olmsted CL, Johnson AM, Kaboli P, Cullen J, Vaughan-Sarrazin MS. Use of palliative care and hospice among surgical and medical specialties in the Veterans Health Administration. JAMA Surg. 2014 Nov;149(11):1169–75.

18. Wu C-C, Hsu T-W, Chang C-M, Lee C-H, Huang C-Y, Lee C-C. Palliative Chemotherapy Affects Aggressiveness of End-of-Life Care. Oncologist. 2016 Jun;21(6):771–7.

19. Gani F, Enumah ZO, Conca-Cheng AM, Canner JK, Johnston FM. Palliative Care Utilization among Patients Admitted for Gastrointestinal and Thoracic Cancers. J Palliat Med. 2018 Apr;21(4):428–37.

20. Wright AA, Zhang B, Keating NL, Weeks JC, Prigerson HG. Associations between palliative chemotherapy and adult cancer patients' end of life care and place of death: prospective cohort study. BMJ. 2014 Mar 4;348:g1219.

21. Pérez-Bracchiglione ISJ. EFFICACY OF SYSTEMIC ONCOLOGICAL TREATMENTS IN PATIENTS WITH ADVANCED, NON-INTESTINAL DIGESTIVE CANCER AT HIGH RISK OF DYING IN THE MIDDLE AND SHORT TERM: EVIDENCE SYNTHESIS [Internet]. 2020. Available from: https://osf.io/7chx6/

22. Pollock M, Fernandes RM, Becker LA, Featherstone R, Hartling L. What guidance is available for researchers conducting overviews of reviews of healthcare interventions? A scoping review and qualitative metasummary [Internet]. Vol. 5, Systematic Reviews. 2016. Available from: http://dx.doi.org/10.1186/s13643-016-0367-5

23. Moher D, Shamseer L, Clarke M, Ghersi D, Liberati A, Petticrew M, et al. Preferred reporting items for systematic review and meta-analysis protocols (PRISMA-P) 2015 statement. Syst Rev. 2015 Jan 1;4:1.

24. Methley AM, Campbell S, Chew-Graham C, McNally R, Cheraghi-Sohi S. PICO, PICOS and SPIDER: a comparison study of specificity and sensitivity in three search tools for qualitative systematic reviews [Internet]. Vol. 14, BMC Health Services Research. 2014. Available from: http://dx.doi.org/10.1186/s12913-014-0579-0

25. Martinic MK, Pieper D, Glatt A, Puljak L. Definition of a systematic review used in overviews of systematic reviews, meta-epidemiological studies and textbooks [Internet]. Vol. 19, BMC Medical Research Methodology. 2019. Available from: http://dx.doi.org/10.1186/s12874-019-0855-0

26. Chin V, Nagrial A, Sjoquist K, O'Connor CA, Chantrill L, Biankin AV, et al. Chemotherapy and radiotherapy for advanced pancreatic cancer [Internet]. Cochrane Database of Systematic Reviews. 2018. Available from: http://dx.doi.org/10.1002/14651858.cd011044.pub2

27. Shea BJ, Reeves BC, Wells G, Thuku M, Hamel C, Moran J, et al. AMSTAR 2: a critical appraisal tool for systematic reviews that include randomised or non-randomised studies of healthcare interventions, or both [Internet]. BMJ. 2017. p. j4008. Available from: http://dx.doi.org/10.1136/bmj.j4008

28. Pieper D, Antoine S-L, Mathes T, Neugebauer EAM, Eikermann M. Systematic review finds overlapping reviews were not mentioned in every other overview [Internet]. Vol. 67, Journal of Clinical Epidemiology. 2014. p. 368–75. Available from: http://dx.doi.org/10.1016/j.jclinepi.2013.11.007

29. Nipp RD, Currow DC, Cherny NI, Strasser F, Abernethy AP, Zafar SY. Best supportive care in clinical trials: review of the inconsistency in control arm design [Internet]. Vol. 113, British Journal of Cancer. 2015. p. 6–11. Available from: http://dx.doi.org/10.1038/bjc.2015.192

30. Guyatt GH, Oxman AD, Vist GE, Kunz R, Falck-Ytter Y, Alonso-Coello P, et al. GRADE: an emerging consensus on rating quality of evidence and strength of recommendations [Internet]. Vol. 336, BMJ. 2008. p. 924–6. Available from: http://dx.doi.org/10.1136/bmj.39489.470347.ad
