# Supplementary material for: Efficacy of systemic oncological treatments in patients with advanced esophageal or gastric cancers at high risk of dying in the middle and short term: an overview of systematic reviews
Source: BMC Cancer. 2021 Jun 16;21:712. doi: 10.1186/s12885-021-08330-5 (PMC8207789; doi:10.1186/s12885-021-08330-5)
Supplement: Supplementary file 3 — Additional file 3. Search strategy. [file 12885_2021_8330_MOESM3_ESM.pdf]

Search strategy for PUBMED

#1	("Gastrointestinal Neoplasms"[Mesh:NoExp] OR "Esophageal Neoplasms"[Mesh] OR "Stomach Neoplasms"[Mesh])
#2	((esophag*[Title] OR oesophag*[Title] OR stomach*[Title] OR gastric*[Title] OR gastroesophag*[Title] OR gastrointestinal*[Title]) AND (cancer*[Title] OR carcinom*[Title] OR neoplasm*[Title] OR tumor*[Title] OR tumour*[Title] OR malignan*[Title] OR adenocar*[Title] OR oncolog*[Title]))
#3	(#1 OR #2)
#4	("Palliative Care"[Mesh] OR "Terminal Care"[Mesh] OR "Neoplasm Metastasis"[Mesh])
#5	(palliative*[Title/Abstract] OR end of life*[Title/Abstract] OR end of live*[Title/Abstract] OR terminal*[Title/Abstract] OR metasta*[Title/Abstract] OR BSC[Title/Abstract] OR supportive care*[Title/Abstract] OR advanced*[Title/Abstract] OR unresect*[Title/Abstract] OR irresect*[Title/Abstract] OR nonresect*[Title/Abstract] OR non resect*[Title/Abstract] OR inopera*[Title/Abstract] OR unopera*[Title/Abstract] OR nonopera*[Title/Abstract] OR non opera*[Title/Abstract] OR non-opera*[Title/Abstract] OR stage IV[Title/Abstract])
#6	(#4 OR #5)
#7	(#3 AND #6)
#8	("Antineoplastic Protocols"[Mesh] OR "Chemoradiotherapy"[Mesh] OR "Induction Chemotherapy"[Mesh] OR "Maintenance Chemotherapy"[Mesh] OR "Consolidation Chemotherapy"[Mesh])
#9	(antineoplastic*[Title/Abstract] OR antineoplasic*[Title/Abstract] OR chemotherap*[Title/Abstract] OR chemoradiotherap*[Title/Abstract] OR radiochemotherap*[Title/Abstract] OR carboplatin*[Title/Abstract] OR cisplatin*[Title/Abstract] OR fluorouracil*[Title/Abstract] OR

	5-FU[Title/Abstract] OR capecitabine*[Title/Abstract] OR docetaxel*[Title/Abstract] OR epirucibin*[Title/Abstract] OR irinotecan*[Title/Abstract] OR oxaliplatin*[Title/Abstract] OR paclitaxel*[Title/Abstract] OR trifluridine*[Title/Abstract] OR tipiracil*[Title/Abstract])
#10	(#8 OR #9)
#11	("Molecular Targeted Therapy"[Mesh] OR "Antibodies, Monoclonal"[Mesh] OR "Cancer Vaccines"[Mesh])
#12	(Target*[Title/Abstract] OR antibod*[Title/Abstract] OR immunotherap*[Title/Abstract] OR vaccine[Title/Abstract] OR vaccines[Title/Abstract] OR vaccination[Title/Abstract] OR HER2[Title/Abstract] OR HER-2[Title/Abstract] OR egfr[Title/Abstract] OR VEGF*[Title/Abstract] OR HGF[Title/Abstract] OR MET[Title/Abstract] OR claudin*[Title/Abstract] OR MMP-9[Title/Abstract][JP1] OR tyrosine kinase inhibit*[Title/Abstract] OR trastuzumab[Title/Abstract] OR bevacizumab[Title/Abstract] OR rilotumumab[Title/Abstract] OR onartuzumab[Title/Abstract] OR ramucirumab[Title/Abstract] OR cetuximab[Title/Abstract] OR panitumumab[Title/Abstract] OR nimotuzumab[Title/Abstract] OR claudiximab[Title/Abstract] OR apatinib[Title/Abstract] OR lapatinib[Title/Abstract] OR regorafenib[Title/Abstract] OR everolimus[Title/Abstract] OR nivolumab[Title/Abstract] OR pembrolizumab[Title/Abstract] OR avelumab[Title/Abstract] OR durvalumab[Title/Abstract] OR ipilimumab[Title/Abstract] OR checkpoint inhibit*[Title/Abstract])
#13	(#11 OR #12)
#14	(#10 OR #13)
#15	(#7 AND #14)
#16	systematic[sb]

#17	(#15 AND #16)
#18	(animals [mh] NOT humans [mh])
#19	(#17 NOT #18)
