# Supplementary material for: Efficacy of systemic oncological treatments in patients with advanced esophageal or gastric cancers at high risk of dying in the middle and short term: an overview of systematic reviews
Source: BMC Cancer. 2021 Jun 16;21:712. doi: 10.1186/s12885-021-08330-5 (PMC8207789; doi:10.1186/s12885-021-08330-5)
Supplement: Supplementary file 4 — Additional file 4. List of excluded reviews and justification for the exclusions. [file 12885_2021_8330_MOESM4_ESM.pdf]

Excluded articles

Study ID	DOI	Reason for exclusion
Abdel-Rahman 2013	10.1007/s10620-013-2872-x	Wrong comparator
Abdel-Rahman 2014	10.1016/j.critrevonc.2013.12.013	Wrong comparator
Abdel-Rahman 2014	10.1586/14737140.2015.985660	Wrong comparator
Abdel-Rahman 2015	10.1586/14737140.2015.978295	Wrong study design
Abdel-Rahman 2016	10.1002/14651858.CD011313.pub2	Wrong intervention
Abdel-Rahman 2017	10.1002/14651858.CD011314.pub2	Wrong intervention
Abdel-Rahman 2017	10.1080/17474124.2017.1264874	Wrong comparator
Abdel-Rahman 2018	10.1002/14651858.CD011746.pub2	Wrong patient population
Abdel-Rahman 2018	10.1002/14651858.CD011746.pub2	Duplicate
Abou-Alfa 2018	10.1200/JCO.2018.36.4-suppl.TPS545	Wrong publication type
Ahmed 2004	10.1002/14651858.CD003445.pub2	Wrong publication type
Ajani 2006	10.1002/cncr.21986	Wrong study design
Al-Batran 2010	10.1002/cncr.25064	Wrong publication type
Ali Abdulnabi Mohamed	No DOI	Wrong publication type
Amdal 2013	10.3109/0284186X.2012.731521	Wrong comparator
Arshad 2013	10.1097/COC.0b013e3182124216	Wrong comparator
Aya El Helali	No DOI	Wrong publication type
Azria 2008	10.1684/bdc.2008.0749	Wrong study design
Baek 2012	10.1007/s10120-011-0114-5	Wrong comparator
Baldo 2015	10.1002/14651858.CD011463	Wrong publication type
Beller 2015	10.1002/14651858.CD010206.pub2	Wrong intervention
Bennetts 2017	10.1016/j.jval.2017.08.107	Wrong comparator
Bian 2019	10.1016/j.ijsu.2018.11.010	Wrong comparator
Brown 2006	No DOI	Wrong study design
Cabalag 2015	10.1007/s10120-014-0388-5	Wrong comparator
Cabibbo 2009	No DOI	Wrong study design
Cabibbo 2010	10.1002/hep.23485	Wrong intervention
Cao 2010	10.1097/MPA.0b013e3181bdc6b8	Wrong comparator
Carter 2015	10.3111/13696998.2015.1066380	Wrong outcomes
Casaretto 2006	10.1590/s0100-879x2006000400002	Wrong publication type
Chan 2013	No DOI	Wrong comparator
Chan 2016	No DOI	Wrong study design
Chan 2019	10.1177/1758835919859990	Wrong study design
Chau 2019	10.1200/JCO.2019.37.4_suppl.128	Wrong publication type
Chen 2013	10.1371/journal.pone.0060320	Wrong comparator
Chen 2018	10.2147/OTT.S157466	Wrong study design
Chen 2019	10.3892/mmr.2018.9638	Wrong comparator
Cherny 2004	10.1093/annonc/mdh928	Wrong study design
Cherny 2009	10.1200/JCO.2009.21.9592	Wrong study design
Chin 2014	No DOI	Wrong publication type
Chin 2018	10.1002/14651858.CD011044.pub2	Wrong patient population
Chintalacheruvu 2017	No DOI	Wrong publication type

Chow 1998	0.1002/14651858.CD001403	Wrong publication type
Chow 2005	10.1016/j.ctrv.2005.07.005	Wrong intervention
Chu 2015	No DOI	Wrong study design
Chua 2006	10.1016/j.bpg.2005.10.003	Wrong study design
Cinar 2017	10.21037/cco.2017.06.13	Wrong study design
Citterio 2018	10.18632/oncotarget.25639	Wrong comparator
Cowley 2017	10.11124/JBISRIR-2016-003108	Wrong comparator
Cuyun Carter 2014	10.1016/j.jval.2014.03.536	Wrong intervention
Dean 2019	10.1200/JCO.2019.37.15_suppl.e15794	Wrong comparator
Desiderio 2017	10.1016/j.ejca.2017.03.030	Wrong comparator
Di Giorgio 2019	10.23736/S0026-4806.19.06081-6	Wrong comparator
Dingle 2005	10.1155/2005/565479	Wrong patient population
Dong 2019	10.5246/JCPS.2019.04.027	Wrong intervention
Duffy 2013	10.1002/hep.26120	Wrong study design
Eckel 2007	10.1038/sj.bjc.6603648	Wrong comparator
Eltawil 2012	10.1111/j.1477-2574.2012.00441.x	Wrong comparator
Facchiano 2012	10.1245/s10434-012-2360-0	Wrong comparator
Fantini 2015	No DOI	Wrong publication type
Faruque 2014	10.1371/journal.pone.0101145	Wrong patient population
Feingold 2017	10.1002/jso.24476	Wrong intervention
Finn 2017	10.1002/hep.29486	Wrong patient population
Fornaro 2015	10.1093/annonc/mdv233.147	Wrong intervention
Gandara-Ladron de Guevara 2014	10.1136/ejhpharm-2013-000436.208	Wrong comparator
Genglong Liu	No DOI	Wrong publication type
Gentile 2019	10.1016/S0016-5085%2819%2940738-5	Wrong intervention
Glimelius 1998	No DOI	Wrong study design
Gollala 2016	No DOI	Wrong comparator
Grassadonia 2018	10.3390/jcm7120542	Wrong patient population
Gresham 2014	10.1186/1471-2407-14-471	Wrong comparator
Hajatdoost 2018	10.3390/medicina54030048	Wrong comparator
Hall 2018	10.1245/s10434-018-6349-1	Wrong comparator
Haun 2017	10.1002/14651858.CD011129.pub2	Wrong intervention
Hill 2006	No DOI	Wrong study design
Hoeben 2016	10.1093/annonc/mdw183	Wrong comparator
Homs 2006		Duplicate
Homs 2010	10.1002/14651858.CD004063.pub3	Duplicate
Hsu 2012	10.1007/s10120-011-0106-5	Wrong comparator
Hu 2017	10.1111/jcpt.12498	Wrong comparator
Huang	No DOI	Wrong comparator
Huang 2017	10.5114/wo.2017.66653	Wrong patient population
Huang 2019	10.1097/MCG.0000000000001175	Wrong patient population
Janmaat 2016	10.1177/2050640616663688	Duplicate
Janmaat 2017	10.1002/14651858.CD004063.pub4	Duplicate
Janowitz 2016	10.1038/bjc.2015.452	Wrong study design

Jen 2015	10.1093/annonc/mdv523.63	Wrong publication type
Jian Yang	No DOI	Wrong publication type
Jiuda Zhao	No DOI	Wrong comparator
Kasuga 2016	No DOI	Wrong publication type
Kasuga 2018	10.1007/s10637-018-0589-6	Wrong comparator
Katz 2019	10.1007/s12029-019-00243-8	Wrong study design
Kerui Wu	No DOI	Wrong comparator
Kim 2013	10.1093/annonc/mdt351	Wrong study design
Kim 2017	10.18632/oncotarget.18314	Wrong study design
Kocher 2010	No DOI	Wrong study design
Kordes 2017	No DOI	Wrong publication type
Kristensen 2016	10.1016/j.critrevonc.2016.01.006	Wrong study design
Lamarca 2014	10.1093/annonc/mdu162	Wrong comparator
Lamarca 2019	10.1093/jnci/djz071	Wrong comparator
Lee 2015	10.1093/annonc/mdv207	Wrong outcomes
Lee 2018	10.1136/bmjopen-2017-017249	Wrong publication type
Lei 2017	10.1097/MD.0000000000006301	Other reasons
Li 2019	10.3389/fonc.2019.00441	Wrong study design
Li 2019	10.1097/MD.0000000000016108	Wrong intervention
Liu 2010	10.1007/s00280-009-1090-x	Wrong publication type
Liu 2014	10.1371/journal.pone.0091124	Wrong comparator
Liu 2016	10.1097/MD.0000000000004993	Wrong comparator
Liu 2019	10.1016/j.jcyt.2019.07.006	Wrong patient population
Llovet 2003	10.1053/jhep.2003.50047	Wrong comparator
Mattiucci 2014	10.1016/j.critrevonc.2013.10.007	Wrong intervention
McNamara 2018	10.1016/j.ejca.2018.09.031	Wrong comparator
Montero 2005	10.1016/S1470-2045%2805%2970094-2	Wrong comparator
Moole 2016	No DOI	Wrong intervention
Moriwaki 2016	10.1038/bjc.2016.83	Wrong study design
Nagrial 2013		Wrong publication type
Niu 2016	10.1097/MD.0000000000005591	Wrong patient population
Nowak 2004	10.1002/14651858.CD001024.pub2	Wrong intervention
Permert 2001	10.1080/02841860151116448	Wrong study design
Qi 2013	10.1002/ijc.27775	Wrong comparator
Qi 2016	10.18632/oncotarget.12102	Wrong study design
Rahma 2013	10.1093/annonc/mdt166	Wrong study design
Roccarina 2017	10.1002/14651858.CD011649.pub2	Wrong patient population
Roccarina 2017	10.1002/14651858.CD011649.pub2	Duplicate
Rossi 2015	10.1002/14651858.CD011568	Wrong publication type
Roviello	No DOI	Wrong publication type
Scartozzi 2007	10.1517/14656566.8.6.797	Wrong study design
Shan 2014	10.1111/ajco.12305	Duplicate
Sharma 2011	10.3748/wjg.v17.i7.867	Wrong study design
Shen 2013	10.1097/MCG.0b013e3182a87cfd	Wrong patient population
Shi 2017	10.1038/s41598-017-05464-0	Wrong comparator

Simonetti 1997	10.1023/a:1008285123736	Wrong study design
Sreedharan 2009	10.1002/14651858.CD005048.pub2	Wrong intervention
Suker 2016	10.1016/S1470-2045(16)00172-8	Wrong comparator
Sultana 2007	10.1038/sj.bjc.6603719	Wrong study design
Sultana 2007	10.1200/JCO.2006.09.2551	Wrong publication type
Sultana 2008	10.1038/sj.bjc.6604436	Wrong comparator
Sultana 2014	10.1002/14651858.CD011044	Wrong patient population
Sun 2013	10.1186/1471-2407-13-577	Wrong intervention
Sun 2017	10.18632/oncotarget.20445	Wrong patient population
Tang 2018	10.1097/MD.0000000000013525	Wrong intervention
Tao Guo 2019	10.7150/jca.32828	Wrong patient population
Tassinari 2015	10.1093/annonc/mdv344.23	Wrong publication type
Tassinari 2015	No DOI	Duplicate
Ter Veer 2016	10.1007/s10120-015-0587-8	Wrong comparator
TerVeer 2018	10.1007/s10120-018-0792-3	Wrong outcomes
The GASTRIC (Global Advanced/Adjuvant Stomach Tumor Research International Collaboration) Group 2013	10.1016/j.ejca.2012.12.016	Wrong study design
Tomita 2016	10.1002/14651858.CD012078	Wrong publication type
Tremblay 2017	10.1093/annonc/mdx369.091	Wrong publication type
Tremblay 2017	No DOI	Duplicate
Tremblay 2017	10.1016/j.jval.2017.08.096	Duplicate
Victor H. F. de Jesus 2020	No DOI	Wrong publication type
Wagner 2005	10.1002/14651858.CD004064.pub2	Duplicate
Wagner 2006	10.1200/JCO.2005.05.0245	Duplicate
Wagner 2010	10.1002/14651858.CD004064.pub3	Duplicate
Wagner 2017	10.1002/14651858.CD004064.pub4	Duplicate
Walma 2016	No DOI	Wrong intervention
Wang 2016	10.2147/DDDT.S105442	Wrong comparator
Wang 2017	10.1002/cam4.1156	Wrong comparator
Wang 2018	10.1097/MD.0000000000010164	Wrong comparator
Weis 2013	10.1002/14651858.CD003046.pub3	Wrong intervention
Whistance 2011	10.1097/SPC.0b013e3283436ecb	Wrong patient population
Yang 2013	10.1371/journal.pone.0057528	Wrong comparator
Yang 2015	10.3978/j.issn.2078-6891.2015.055	Wrong patient population
Yerasi 2016	No DOI	Wrong outcomes
Yip 2006	10.1002/14651858.CD002093.pub2	Wrong patient population
Zagouri 2013	10.1097/MPA.0b013e31827aedef	Wrong comparator
Zhang 2010	10.1097/CAD.0b013e3283350e26	Wrong study design
Zhang 2012	10.1016/S1499-3872(12)60209-4	Wrong patient population
Zhang 2018	10.1093/annonc/mdy282.111	Wrong comparator
Zheng 2017	10.1097/MD.0000000000006884	Wrong study design
Zhu 2015	10.1371/journal.pone.0128616	Wrong comparator
Zhuang 2013	10.1371/journal.pone.0061361	Wrong comparator

Ziyu Li	No DOI	Wrong publication type
Zou 2011	10.3881/j.issn.1000-503X.2011.01.011	Wrong patient population
