# Supplementary material for: Efficacy of systemic oncological treatments in patients with advanced esophageal or gastric cancers at high risk of dying in the middle and short term: an overview of systematic reviews
Source: BMC Cancer. 2021 Jun 16;21:712. doi: 10.1186/s12885-021-08330-5 (PMC8207789; doi:10.1186/s12885-021-08330-5)
Supplement: Supplementary file 5 — Additional file 5. Further characteristics of included systematic reviews. [file 12885_2021_8330_MOESM5_ESM.pdf]

Appendix 5. Further characteristics of Systematic Reviews

Author (year)	Aim	Conclusion
**Iacovelli 2014**	To estimate the effect of second-line treatment of gastric cancer and to analyze the differential role of chemotherapy or targeted agents.<br><br>Also, to investigate if different strategies have the same role in patients with different performance status, with the intent to find the best strategy for second-line treatment of this tumor.	This study confirms a significant benefit in terms of OS when active second-line treatments are administered to patients with advanced gastric cancer after failure of a previous line of therapy even in patients with impaired performance status.<br><br>If the lack of difference between chemotherapy agents was reported by other studies, we suggest a lack of difference between chemotherapy and ramucirumab. Further studies are urgently required to better understand the clinical or molecular characteristics for patient' selection.
**Veer 2016**	To summarize the evidence regarding all possible second- or third-line treatments in patients with advanced esophagogastric cancer.	This review indicates that, given the survival benefit in a phase III study setting, ramucirumab plus taxane is the preferred second-line treatment. Taxane or irinotecan monotherapy are alternatives, although the absolute survival benefit was limited. In the third-line setting, apatinib monotherapy is preferred.
**Wang 2016**	To assess the efficacy of targeted agents (TAs) in the treatment of elderly patients with advanced gastric cancer (AGC).	This is the first meta-analysis specifically as sessing the efficacy of adding TAs to therapies in elderly AGC patients. The results of our study suggest that the use of TAs in elderly AGC patients offers an improved OS which could be ascribed to AIs and HER-2 agents. With present available data from randomized clinical trials, the use of anti-EGFR agents in elderly AGC patients could not be recommended.
**Chan a 2017**	To evaluate the overall effect of anti-angiogenic agents, in combination with chemotherapy and as monotherapy, in the treatment of metastatic oesophago-gastric cancer (mOGC), with respect to the outcomes of overall survival, progression-free survival, response rate, toxicity measures and quality of life.	The addition of AAs to standard therapy in mOGC improves OS. Improved efficacy was only observed in 2nd- or 3rd-line setting and not in 1st-line setting. Consistent OS benefit was present across all geographical regions. This benefit is at the expense of increased overall toxicity.

**Chan b 2017**	To evaluate the efficacy and toxicity of third-line treatment including both chemotherapy and targeted agents.	The results suggested a statistically significant but modest clinical benefit in favour of TLT, in the cost of more toxicities. QOL data after failure of second-line therapy is scarce and incomplete. Truthful communication with patients and carers is of utmost importance before starting TLT. Future research should focus on the combination and sequencing of different anti-cancer agents for advanced gastric cancer to maximise the efficacy and minimise the toxicities.
**Harvey 2017**	To compare different interventions for treatment of second-line advanced gastric cancer (AGC) using more complex methods to estimate relative efficacy, fitting various parametric models and to compare results to those published adopting conventional methods of synthesis.	Whilst the approach adopted in this paper does not adjust for differences in trial patient populations and is particularly data-in-tensive, use of such sophisticated methods of evidence synthesis may be more informative for subsequent cost-effectiveness modelling and may have greater impact when considering an indication where observed data is particularly immature or survival prospects are more positive, which may then lead to more informative decision-making for drug reimbursement.
**Janmat 2017**	To assess the effects of cytostatic or targeted therapy for treating esophageal or gastroesophageal junction cancer with palliative intent.	People who receive more chemotherapeutic or targeted therapeutic agents have an increased overall survival compared to people who receive less. These agents, administered as both first-line or second-line treatments, also led to better overall survival than best supportive care. With the exception of ramucirumab, it remains unclear which other individual agents cause the survival benefit. Although treatment-associated toxicities of grade 3 or more occurred more frequently in arms with an additional chemotherapy or targeted therapy agent, there is no evidence that palliative chemotherapy and/or targeted therapy decrease quality of life. Based on this meta-analysis, palliative chemotherapy and/or targeted therapy can be considered standard care for esophageal and gastroesophageal junction carcinoma.
**Wagner 2017**	To assess the efficacy of chemotherapy versus	Chemotherapy improves survival (by an additional 6.7 months) in

	best supportive care (BSC), combination versus single-agent chemotherapy and different chemotherapy combinations in advanced gastric cancer.	comparison to BSC, and combination chemotherapy improves survival (by an additional month) compared to single-agent 5-FU. Testing all patients for HER-2 status may help to identify patients with HER-2-positive tumours, for whom, in the absence of contraindications, trastuzumab in combination with capecitabine or 5-FU in combination with cisplatin has been shown to be beneficial. For HER-2 negative people, all different two-and three-drug combinations including irinotecan, docetaxel, oxaliplatin or oral 5-FU prodrugs are valid treatment options for advanced gastric cancer, and consideration of the side effects of each regimen is essential in the treatment decision. Irinotecan-containing combinations and docetaxel-containing combinations (in which docetaxel was added to a single-agent or two-drug (platinum/5-FUcombination) show significant survival benefits in the comparisons studied above. Furthermore, docetaxel-containing three-drug regimens have increased response rates, but the advantages of the docetaxel-containing three-drug combinations (DCF, FLO-T) are counterbalanced by increased toxicity. Additionally, oxaliplatin-containing regimens demonstrated a benefit in OS as compared to the same regimen containing cisplatin, and there is a modest survival improvement of S-1 compared to 5-FU-containing regimens.
**Wang 2017**	To perform a systematic review and meta-analysis of Phase III randomized controlled trials (RCTs) to determine the incidence and risk of severe adverse events (AEs) with molecular targeted agents (MTAs) in advanced/metastatic gastric cancer (GC) patients.	In conclusion, this is the first meta-analysis that specifically assessed the severe and fatal toxicities of adding MTAs to therapies in the treatment of GC patients. The results of our study suggest that the addition of MTAs to therapies in GC significantly increases the risk of developing severe AEs, but not for FAEs. Additionally, the most common causes of FAEs with MTAs were infections, gastrointestinal hemorrhage, and arterial thromboembolic events, respectively.
**Xie 2017**	To evaluate the performance of different targeted	In conclusion, trastuzumab was recommended as the optimal

	drugs used in combination with chemotherapy and try to find out the most effective one/ones for patients with advanced gastric cancer.	targeted agent combined with chemotherapy for gastric cancer patients.
**Zhu 2017**	To conduct a network meta-analysis to create a network of therapeutic regimens for advanced gastric cancer in the second-line setting, and to determine their relative efficacy.	This is the first network meta-analysis to compare all second-line regimens reported in phase III gastric cancer trials. The results suggest the paclitaxel plus ramucirumab combination is the most effective therapy and should be the reference regimen for future comparative trials.
**Liu 2018**	This study aimed to serve as the first systematic review to assess their safety and efficacy according to biochemical characteristics of targeting VEGFR drugs in gastric cancer.	VEGFR drugs were effective targeted therapy in advanced or metastatic gastric cancer, and its toxicity is within a controllable range. VEGFR-Ab drugs were more effective than VEGFR-TKI drugs in terms of the OS, PFS and PDR of gastric cancer patients with little toxicity.
**Zhao 2018**	This study evaluates the efficacy and safety of targeted agents for Advanced gastric cancer	Apatinib, regorafenib, and rilotumumab improved patient PFS and OS. When combined with chemotherapy, ramucirumab and rilotumumab had high efficacy but low tolerability, and bevacizumab had moderate efficacy and tolerability for PFS. Without chemotherapy, ramucirumab and regorafenib had relatively high therapeutic efficacy tolerability for PFS.
**Chen 2019**	To evaluate the efficacy and safety of ICI in G/GEJ cancer.	ICI treatment could improve some but not all survival endpoints to advanced or metastatic G/GEJ cancer patients suggesting modest benefit and less adverse reactions. Anti-PD-1/PD-L1 therapy was more effective to PD-L1+, MSI-H, EBV+, or high tumor mutational burden patients.
**van Kleef 2020**	To examine the impact of systemic therapy on HRQoL of patients with advanced esophagogastric cancer more comprehensively using meta-analysis to answer the following four research questions: What are the most affected	Patients reported impaired GHS at baseline and generally remained stable over time. Anthracycline-based triplets and fluoropyrimidine-based doublets without cisplatin may be preferable first-line treatment options regarding HRQoL for HER2-negative disease. Taxanes and targeted agents could

	disease-related functions and symptoms before start of<br>treatment in the first-line and beyond first-line treatment setting? What is the course of HRQoL over time? Which chemotherapy regimens show better HRQoL over comparator regimens? Is there a relationship between HRQoL and OS?	provide HRQoL benefit beyond the first line compared with best supportive care.
**Wallis 2019**	To perform an updated, comprehensive meta-analysis that assesses the efficacy of immunotherapy in advanced cancers according to patient sex.	In this contemporary meta-analysis of all available immunotherapy clinical trials across all disease sites, we found no difference in immunotherapy efficacy or OS between women and men. Contrary to findings of a previous analysis, we found no evidence that sex should be considered when deciding whether to offer immunotherapy to patients with advanced cancers.
