# Supplementary material for: Efficacy of systemic oncological treatments in patients with advanced esophageal or gastric cancers at high risk of dying in the middle and short term: an overview of systematic reviews
Source: BMC Cancer. 2021 Jun 16;21:712. doi: 10.1186/s12885-021-08330-5 (PMC8207789; doi:10.1186/s12885-021-08330-5)
Supplement: Supplementary file 6 — Additional file 6. References RCTs. [file 12885_2021_8330_MOESM6_ESM.pdf]

Appendix 6. References 19 RCTs included in systematic reviews

Bang, Y.-J., Ruiz, E. Y., Van Cutsem, E., Lee, K.-W., Wyrwicz, L., Schenker, M., Alsina, M., Ryu, M.-H., Chung, H.-C., Evesque, L., Al-Batran, S.-E., Park, S. H., Lichinitser, M., Boku, N., Moehler, M. H., Hong, J., Xiong, H., Hallwachs, R., Conti, I., & Taieb, J. (2018). Phase III, randomised trial of avelumab versus physician's choice of chemotherapy as third-line treatment of patients with advanced gastric or gastro-oesophageal junction cancer: primary analysis of JAVELIN Gastric 300. *Annals of Oncology: Official Journal of the European Society for Medical Oncology / ESMO*, *29*(10), 2052–2060.

Bramhall, S. R., Hallissey, M. T., Whiting, J., Scholefield, J., Tierney, G., Stuart, R. C., Hawkins, R. E., McCulloch, P., Maughan, T., Brown, P. D., Baillet, M., & Fielding, J. W. L. (2002). Marimastat as maintenance therapy for patients with advanced gastric cancer: a randomised trial. *British Journal of Cancer*, *86*(12), 1864–1870.

Demetri, G. D., Reichardt, P., Kang, Y.-K., Blay, J.-Y., Rutkowski, P., Gelderblom, H., Hohenberger, P., Leahy, M., von Mehren, M., Joensuu, H., Badalamenti, G., Blackstein, M., Le Cesne, A., Schöffski, P., Maki, R. G., Bauer, S., Nguyen, B. B., Xu, J., Nishida, T., … Casali, P. G. (2013). Efficacy and safety of regorafenib for advanced gastrointestinal stromal tumours after failure of imatinib and sunitinib (GRID): an international, multicentre, randomised, placebo-controlled, phase 3 trial. *The Lancet*, *381*(9863), 295–302.

Dutton, S. J., Ferry, D. R., Blazeby, J. M., Abbas, H., Dahle-Smith, A., Mansoor, W., Thompson, J., Harrison, M., Chatterjee, A., Falk, S., Garcia-Alonso, A., Fyfe, D. W., Hubner, R. A., Gamble, T., Peachey, L., Davoudianfar, M., Pearson, S. R., Julier, P., Jankowski, J., … Petty, R. D. (2014). Gefitinib for oesophageal cancer progressing after chemotherapy (COG): a phase 3, multicentre, double-blind, placebo-controlled randomised trial. *The Lancet Oncology*, *15*(8), 894–904.

Ford, H. E. R., Marshall, A., Bridgewater, J. A., Janowitz, T., Coxon, F. Y., Wadsley, J., Mansoor, W., Fyfe, D., Madhusudan, S., Middleton, G. W., Swinson, D., Falk, S., Chau, I., Cunningham, D., Kareclas, P., Cook, N., Blazeby, J. M., & Dunn, J. A. (2014). Docetaxel versus active symptom control for refractory oesophagogastric adenocarcinoma (COUGAR-02): an open-label, phase 3 randomised controlled trial. *The Lancet Oncology*, *15*(1), 78–86.

Fuchs, C. S., Tomasek, J., Yong, C. J., Dumitru, F., Passalacqua, R., Goswami, C., Safran, H., dos Santos, L. V., Aprile, G., Ferry, D. R., Melichar, B., Tehfe, M., Topuzov, E., Zalcberg, J. R., Chau, I., Campbell, W., Sivanandan, C., Pikiel, J., Koshiji, M., … Tabernero, J. (2014). Ramucirumab monotherapy for previously treated advanced gastric or gastro-oesophageal junction adenocarcinoma (REGARD): an international, randomised, multicentre, placebo-controlled, phase 3 trial. *The Lancet*, *383*(9911), 31–39.

Glimelius, B., Ekström, K., Hoffman, K., Graf, W., Sjödén, P.-O., Haglund, U., Svensson, C., Enander, L.-K., Linné, T., Sellsröm, H., & Heuman, R. (1997). Randomized comparison between chemotherapy plus best supportive care with best supportive care in advanced gastric cancer. *Annals of Oncology: Official Journal of the European Society for Medical Oncology / ESMO*, *8*(2), 163–168.

Kang, J. H., Lee, S. I., Lim, D. H., Park, K.-W., Oh, S. Y., Kwon, H.-C., Hwang, I. G., Lee, S.-C., Nam, E., Shin, D. B., Lee, J., Park, J. O., Park, Y. S., Lim, H. Y., Kang, W. K., & Park, S. H. (2012). Salvage Chemotherapy for Pretreated Gastric Cancer: A Randomized Phase III Trial Comparing Chemotherapy Plus Best Supportive Care With Best Supportive Care Alone. *Journal of Clinical Orthodontics: JCO*, *30*(13), 1513–1518.

Kang, Y.-K., Boku, N., Satoh, T., Ryu, M.-H., Chao, Y., Kato, K., Chung, H. C., Chen, J.-S.,

Muro, K., Kang, W. K., Yeh, K.-H., Yoshikawa, T., Oh, S. C., Bai, L.-Y., Tamura, T., Lee, K.-W., Hamamoto, Y., Kim, J. G., Chin, K., … Chen, L.-T. (2017). Nivolumab in patients with advanced gastric or gastro-oesophageal junction cancer refractory to, or intolerant of, at least two previous chemotherapy regimens (ONO-4538-12, ATTRACTION-2): a randomised, double-blind, placebo-controlled, phase 3 trial. *The Lancet*, *390*(10111), 2461–2471.

Levard, H., Pouliquen, X., Hay, J.-M., Fingerhut, A., Langlois-Zantain, O., Huguier, M., Lozach, P., & Testart, J. (2003). 5-Fluorouracil and cisplatin as palliative treatment of advanced oesophageal squamous cell carcinoma: A multicentre randomised controlled trial. *The European Journal of Surgery = Acta Chirurgica*, *164*(11), 849–857.

Li, J., Qin, S., Xu, J., Guo, W., Xiong, J., Bai, Y., Sun, G., Yang, Y., Wang, L., Xu, N., Cheng, Y., Wang, Z., Zheng, L., Tao, M., Zhu, X., Ji, D., Liu, X., & Yu, H. (2013). Apatinib for Chemotherapy-Refractory Advanced Metastatic Gastric Cancer: Results From a Randomized, Placebo-Controlled, Parallel-Arm, Phase II Trial. *Journal of Clinical Orthodontics: JCO*, *31*(26), 3219–3225.

Li, J., Qin, S., Xu, J., Xiong, J., Wu, C., Bai, Y., Liu, W., Tong, J., Liu, Y., Xu, R., Wang, Z., Wang, Q., Ouyang, X., Yang, Y., Ba, Y., Liang, J., Lin, X., Luo, D., Zheng, R., … Yu, H. (2016). Randomized, Double-Blind, Placebo-Controlled Phase III Trial of Apatinib in Patients With Chemotherapy-Refractory Advanced or Metastatic Adenocarcinoma of the Stomach or Gastroesophageal Junction. *Journal of Clinical Oncology: Official Journal of the American Society of Clinical Oncology*, *34*(13), 1448–1454.

Murad, A. M., Santiago, F. F., Petroianu, A., Rocha, P. R., Rodrigues, M. A., & Rausch, M. (1993). Modified therapy with 5-fluorouracil, doxorubicin, and methotrexate in advanced gastric cancer. *Cancer*, *72*(1), 37–41.

Nicolaou, N., & Conlan, A. A. (1982). Cyclophosphamide, doxorubicin and celestin intubation for inoperable oesophageal carcinoma. *South African Medical Journal = Suid-Afrikaanse Tydskrif Vir Geneeskunde*, *61*(12), 428–431.

Ohtsu, A., Ajani, J. A., Bai, Y.-X., Bang, Y.-J., Chung, H.-C., Pan, H.-M., Sahmoud, T., Shen, L., Yeh, K.-H., Chin, K., Muro, K., Kim, Y. H., Ferry, D., Tebbutt, N. C., Al-Batran, S.-E., Smith, H., Costantini, C., Rizvi, S., Lebwohl, D., & Van Cutsem, E. (2013). Everolimus for Previously Treated Advanced Gastric Cancer: Results of the Randomized, Double-Blind, Phase III GRANITE-1 Study. *Journal of Clinical Orthodontics: JCO*, *31*(31), 3935–3943.

Pavlakis, N., Sjoquist, K. M., Tsobanis, E., Martin, A., Kang, Y.-K., Bang, Y.-J., O'Callaghan, C. J., Tebbutt, N. C., Rha, S. Y., Lee, J., Cho, J. Y., Lipton, L. R., Burnell, M. J., Alcindor, T., Strickland, A., Wong, M., Kim, J. W., Simes, J., Zalcberg, J. R., & Goldstein, D. (2015). INTEGRATE: A randomized phase II double-blind placebo-controlled study of regorafenib in refractory advanced oesophagogastric cancer (AOGC)—A study by the Australasian Gastrointestinal Trials Group (AGITG), first results. *Journal of Clinical Orthodontics: JCO*, *33*(3_suppl), 9–9.

Pyrhönen, S., Kuitunen, T., Nyandoto, P., & Kouri, M. (1995). Randomised comparison of fluorouracil, epidoxorubicin and methotrexate (FEMTX) plus supportive care with supportive care alone in patients with non-resectable gastric cancer. *British Journal of Cancer*, *71*(3), 587–591.

Scheithauer W, Kornek G, Hejna M, Depisch D, Raderer M, Huber H. (1996). Palliative chemotherapy versus best supportive care in patients with metastatic gastric cancer: a randomized trial. *Annals of Hematology*, *73*(Suppl 2), A181.

Thuss-Patience, P. C., Kretzschmar, A., Bichev, D., Deist, T., Hinke, A., Breithaupt, K., Dogan, Y., Gebauer, B., Schumacher, G., & Reichardt, P. (2011). Survival advantage for

irinotecan versus best supportive care as second-line chemotherapy in gastric cancer – A randomised phase III study of the Arbeitsgemeinschaft Internistische Onkologie (AIO). *European Journal of Cancer*, *47*(15), 2306–2314.
