# Supplementary material for: Efficacy of systemic oncological treatments in patients with advanced esophageal or gastric cancers at high risk of dying in the middle and short term: an overview of systematic reviews
Source: BMC Cancer. 2021 Jun 16;21:712. doi: 10.1186/s12885-021-08330-5 (PMC8207789; doi:10.1186/s12885-021-08330-5)
Supplement: Supplementary file 7 — Additional file 7. GRADE. [file 12885_2021_8330_MOESM7_ESM.pdf]

Summary of findings:

Chemotherapy compared to best supportive care or placebo for advanced gastric cancer

Patient or population: advanced gastric cancer

Intervention: chemotherapy

Comparison: best supportive care or placebo

Outcome<br>№ of participants<br>(studies)	Relative effect<br>(95% CI)	Anticipated absolute effects (95% CI)			Certainty	What happens
				Difference		
		High				
Overall survival<br>№ of participants: 426<br>(5 RCTs)	**HR 0.44**<br>(0.33 to 0.58)<br>[Overall survival]	25.0%	**11.9%**<br>(9.1 to 15.4)	**13.1% fewer**<br>(15,9 fewer to 9,6 fewer)	⊕⊕◯◯<br>LOW [a,b]	The evidence is uncertain about the effect of chemotherapy on overall survival at 12 months
Quality of life<br>№ of participants: (0 studies)	No studies were found that reported quality of life				-	
Functional status<br>№ of participants: (0 studies)	No studies were found that reported functional status				-	
Toxicity<br>№ of participants: (5 RCTs)	Studies reported that between 12% to 32,7% of the participants from the CT group experienced toxicity.				⊕⊕⊕◯<br>MODERATE [a]	We are moderately confident that chemotherapy probably increases toxicity.

***The risk in the intervention group** (and its 95% confidence interval) is based on the assumed risk in the comparison group and the **relative effect** of the intervention (and its 95% CI).

CI: Confidence interval; **HR:** Hazard Ratio

GRADE Working Group grades of evidence
High certainty: We are very confident that the true effect lies close to that of the estimate of the effect
Moderate certainty: We are moderately confident in the effect estimate: The true effect is likely to be close to the estimate of the effect, but there is a possibility that it is substantially different
Low certainty: Our confidence in the effect estimate is limited: The true effect may be substantially different from the estimate of the effect
Very low certainty: We have very little confidence in the effect estimate: The true effect is likely to be substantially different from the estimate of effect

Explanations

a. We downgraded one level due to serious concerns about risk of bias on the quality of attrition and detection assesment

b. We downgraded one level due to the available evidence comes from a number of small studies, most of which have been commercially funded

Summary of findings:

Chemotherapy compared to best supportive care or placebo for advanced esophageal cancer

Patient or population: advanced esophageal cancer

Intervention: chemotherapy

Comparison: best supportive care or placebo

Outcome<br>№ of participants<br>(studies)	Relative effect<br>(95% CI)	Anticipated absolute effects (95% CI)			Certainty	What happens
				**Difference**		
		**High**				
Overall survival<br>№ of participants: 118<br>(2 RCTs)	**HR 0.77**<br>(0.63 to 0.94)	73.0%	**63.5%**<br>(56.2 to 70.8)	**9.5% fewer**<br>(16,8 fewer to 2,2 fewer)	⨁◯◯◯<br>VERY LOW [a,b,c]	The evidence is very uncertain about the effect of chemotherapy on overall survival at 12 months.
Quality of life<br>№ of participants: (studies)	No studies were found that reported quality of life				-	
Functional status<br>№ of participants: (studies)	No studies were found that reported functional status				-	
Toxicity<br>№ of participants: (studies)	No studies were found that reported toxicity				-	

***The risk in the intervention group** (and its 95% confidence interval) is based on the assumed risk in the comparison group and the **relative effect** of the intervention (and its 95% CI).

CI: Confidence interval; **HR:** Hazard Ratio

GRADE Working Group grades of evidence
High certainty: We are very confident that the true effect lies close to that of the estimate of the effect
Moderate certainty: We are moderately confident in the effect estimate: The true effect is likely to be close to the estimate of the effect, but there is a possibility that it is substantially different
Low certainty: Our confidence in the effect estimate is limited: The true effect may be substantially different from the estimate of the effect
Very low certainty: We have very little confidence in the effect estimate: The true effect is likely to be substantially different from the estimate of effect

Explanations

a. We downgraded one level due to serious concerns about unclear risk of bias on many domains

b. We downgraded one level due to imprecision. Small sample size.

c. We downgraded one level because available evidence comes from a number of small studies.

Summary of findings:

Inmunotherapy compared to best supportive care or placebo for advanced gastric cancer (including GEJ)

Patient or population: advanced gastric cancer (including GEJ)

Intervention: inmunotherapy

Comparison: best supportive care or placebo

Outcome<br>№ of participants<br>(studies)	Relative effect<br>(95% CI)	Anticipated absolute effects (95% CI)			Certainty	What happens
				Difference		
		High				
Overall survival<br>№ of participants:<br>(2 RCTs)	**HR 0.70**<br>(0.52 to 0.93)	25.0%	**18.2%**<br>(13.9 to 23.5)	**6.8% fewer**<br>(11,1 fewer to 1,5 fewer)	⊕◯◯◯<br>VERY LOW [a,b,c]	
Quality of life<br>№ of participants:<br>(studies)	No studies were found that reported at quality of life				-	
Functional status<br>№ of participants:<br>(studies)	No studies were found that reported at functional status				-	
Toxicity<br>№ of participants:<br>(studies)	No studies were found that reported at toxicity				-	

***The risk in the intervention group** (and its 95% confidence interval) is based on the assumed risk in the comparison group and the **relative effect** of the intervention (and its 95% CI).

CI: Confidence interval; **HR:** Hazard Ratio

GRADE Working Group grades of evidence
High certainty: We are very confident that the true effect lies close to that of the estimate of the effect
Moderate certainty: We are moderately confident in the effect estimate: The true effect is likely to be close to the estimate of the effect, but there is a possibility that it is substantially different
Low certainty: Our confidence in the effect estimate is limited: The true effect may be substantially different from the estimate of the effect
Very low certainty: We have very little confidence in the effect estimate: The true effect is likely to be substantially different from the estimate of effect

Explanations

a. We downgraded one level due to serious concerns about risk of bias on the quality of blinding

b. We downgraded two levels due to very serious imprecision. Large 95% confidence interval, few events and small sample size

c. We downgraded one level because available evidence comes from a number of small studies.

Summary of findings:

Biological therapy compared to best supportive care or placebo for advanced gastric cancer (including GEJ)

Patient or population: advanced gastric cancer (including GEJ)

Intervention: biological therapy

Comparison: best supportive care or placebo

Outcome<br>№ of participants<br>(studies)	Relative effect<br>(95% CI)	Anticipated absolute effects (95% CI)			Certainty	What happens
				Difference		
		High				
Overall survival<br>№ of participants: 1157<br>(6 RCTs)	**HR 0.64**<br>(0.51 to 0.80)	25.0%	**16.8%**<br>(13.6 to 20.6)	**8.2% fewer**<br>(11,4 fewer to 4,4 fewer)	⊕⊕⊕◯<br>MODERATE [a]	We are moderately confident that biological therapy probably improves overall survival.
Quality of life<br>№ of participants: (9 RCTs)	HRQoL benefit was observed for taxane monotherapy and several targeted agents over best supportive care beyond the first line. Studies reporting no difference in global QoL investigated apatinib, ramucirumab and bevacizumab.				⊕◯◯◯<br>VERY LOW [a,b,c]	The evidence is very uncertain about the effect of biological therapy treatment on quality of life.
Functional status<br>№ of participants: (0 studies)	No studies were found that looked at functional status				-	
Toxicity<br>№ of participants: (1 RCT)	Marimastat increases risk of severe AEs vs placebo. (OR 1,46 IC 95% 0,80 - 2,67)				⊕◯◯◯<br>VERY LOW [a,b,c]	

***The risk in the intervention group** (and its 95% confidence interval) is based on the assumed risk in the comparison group and the **relative effect** of the intervention (and its 95% CI).

CI: Confidence interval; **HR:** Hazard Ratio

GRADE Working Group grades of evidence
High certainty: We are very confident that the true effect lies close to that of the estimate of the effect
Moderate certainty: We are moderately confident in the effect estimate: The true effect is likely to be close to the estimate of the effect, but there is a possibility that it is substantially different
Low certainty: Our confidence in the effect estimate is limited: The true effect may be substantially different from the estimate of the effect
Very low certainty: We have very little confidence in the effect estimate: The true effect is likely to be substantially different from the estimate of effect

Explanations

a. We downgraded one level due to serious imprecision. The 95% confidence intervals are large and small sample sizes of each study.

b. We downgraded one level due to serious concerns about risk of bias on the quality of randomisation, and blinding.

c. We downgraded one level because available evidence comes from only one small study.
